# Six New Phenolic Compounds in Ethyl Acetate Extract of Tall Gastrodia Tuber (Tianma) with Four Compounds Screened Preliminarily for Cytoprotective Effects Against Excitotoxicity

**DOI:** 10.3390/ph19071068

**Published:** 2026-07-10

**Authors:** Han Yu, Shiwei Huang, Jiahao Wang, Yaogamo Jile, Yuxin Gao, Songling Li, Ningjing Li, Aimin Zhong, Kaifeng Hu, Guanghua Lu

**Affiliations:** 1 School of Pharmacy, Chengdu University of Traditional Chinese Medicine, Chengdu 611137, China; 2 Chinese Medicine Germplasm Resources Innovation and Effective Uses Key Laboratory of Sichuan Province, Chengdu 611137, China; 3 Research Institute of Chinese Medicines as Drug & Food, Chengdu University of Traditional Chinese Medicine, Chengdu 611137, China; 4 Sichuan Chijian Traditional Chinese Medicine Technology Co., Ltd., Guangyuan 628005, China; 5 Innovative Institute of Chinese Medicine and Pharmacy, Chengdu University of Traditional Chinese Medicine, Chengdu 611137, China

**Keywords:** *Gastrodia elata*, Tall Gastrodia Tuber, chemical constituent, phenolic compounds, phenolic nucleoside, glutamate-induced excitotoxicity

## Abstract

**Background/Objectives:** Tall Gastrodia Tuber (tuber of *Gastrodia elata*, Tianma) is an important herb with therapeutic effects on multiple central nervous system diseases. Although some chemical compounds with their bioactivities have been reported, there are still unrevealed bioactive compounds. This study focuses on discovering new compounds with pharmacological effects in Tianma. **Methods:** Compounds were isolated from ethyl acetate extract of Tianma by column chromatography and semi-preparative HPLC. Their chemical structures were elucidated by spectroscopic analysis including two-dimensional NMR (DEPT90/135, COSY, HSQC, HMBC), HR-ESI-MS, and IR, compared with reported data. Meanwhile, the protective effects against glutamate-induced excitotoxicity of two phenolic glucosides and two rare phenolic nucleosides were screened by a HT-22 cell model. **Results:** Fourteen compounds were identified, comprising six new natural products and eight known compounds. The six new compounds were named gastrotribenzyloside A (**1**), gastrotribenzyloside B (**2**), gastrotribenzyloside C (**3**), gastrotetrabenzyloside D (**4**), gastronucleoside B (**5**) and gastronucleoside C (**6**). Compounds **1**–**4** were phenolic glucosides. Compounds **5** and **6** were the first discovery of phenolic nucleosides substituted by multiple benzyl groups in Tianma. A bioactive experiment indicated that the two phenolic glucosides **1** and **2** did not exhibit protective effects against glutamate-induced excitotoxicity of HT-22 cells, while both of the phenolic nucleosides **5** and **11** significantly relieved the cell viability reduction caused by glutamate. **Conclusions:** Six new phenolic glucosides and phenolic nucleosides are discovered in Tianma. These phenolic nucleosides are potential bioactive components exhibiting cytoprotective effects against excitotoxicity. They deserve further in vivo studies to verify their bioactivity and investigate the mechanism.

## 1. Introduction

Tall Gastrodia Tuber (the tubers of *Gastrodia elata* Blume, Tianma in Chinese transliteration) is a precious herb with a long history of thousands of years [1]. Since the 1980s, mature cultivation techniques for Tianma have been popularized, transforming Tianma into an affordable herb [2,3]. Traditionally, it is usually used to treat diseases related to epilepsy and obnubilation. Modern studies have demonstrated that Tianma can significantly ameliorate diseases of the central nervous system, liver, heart, etc. [4,5,6]. In particular, its therapeutic effects against epilepsy, depression and memory disorder may help to solve health problems resulting from ageing populations and modern life stress [7,8,9,10]. Therefore, Tianma has been commonly applied as both medicine and food [11]. The ethyl acetate extract of Tianma exhibited significant ameliorative effects on models of various neurological disorders in recent studies [12,13]. Although some bioactive phenolic compounds have been discovered in this chemical fraction, new chemical constituents with their bioactivities remain unknown [14,15]. So, it is important to further discover new chemical constituents and bioactivities to promote the full utilization of Tianma.

Glutamate-induced excitotoxicity is an important mechanism of multiple central nervous system diseases, e.g., epilepsy, Parkinson’s disease and vascular dementia [16]. Modern studies have shown that the ethyl acetate extract of Tianma and some bioactive constituents exhibit significant protective effects on such diseases [17,18]. However, few bioactive studies have been conducted on these constituents, especially compounds of rare categories, e.g., phenolic nucleosides, polyphenols and phenolic lactates [19,20].

**Figure 1:**
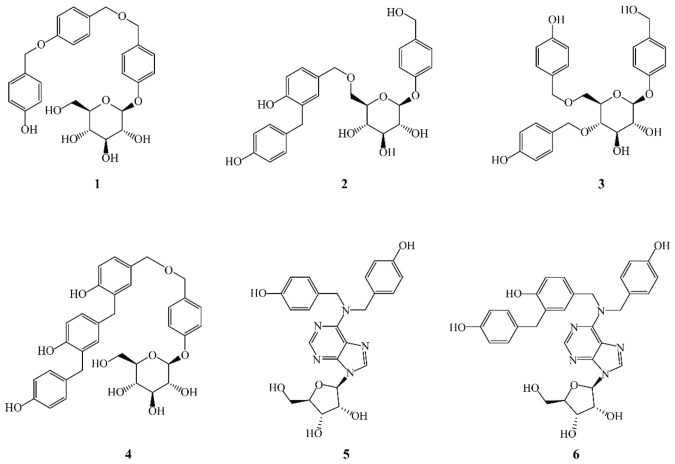
Chemical structures of six new compounds isolated from Tianma: (**1**) gastrotribenzyloside A; (**2**) gastrotribenzyloside B; (**3**) gastrotribenzyloside C; (**4**) gastrotetrabenzyloside D; (**5**) gastronucleoside B; (**6**) gastronucleoside C.

The present study focused on investigating the chemical constituents in the ethyl acetate extract of Tianma and preliminarily evaluating bioactivity. A Tianma sample was extracted with ethanol and water respectively by reflux. The extract was separated by various solvents. Then, the ethyl acetate extract was separated by column chromatography and semi-preparative HPLC to yield 14 pure compounds. Their chemical structures were elucidated based on UV, FT-IR, MS, NMR spectra, etc. These compounds included six new natural phenolic compounds, which were named gastrotribenzyloside A (**1**), gastrotribenzyloside B (**2**), gastrotribenzyloside C (**3**), gastrotetrabenzyloside D (**4**), gastronucleoside B (**5**) and gastronucleoside C (**6**) (Figure 1). Meanwhile, eight known compounds were obtained, i.e., 2-[4-hydroxy-3-(4-hydroxybenzyl) benzyl]-4-methoxymethyl phenol (**7**), 6′,7-di-*O*-(4-hydroxybenzyl) gastrodin (**8**), 4-hydroxy-3-(4-hydroxybenzyl) benzyl methyl ether (**9**), 6′,7-di-*O*-(4-hydroxybenzyl) gastrodin (**10**), *N*^6^-(4-hydroxybenzyl) adenosine (**11**), 2′-*O*-(4-hydroxybenzyl) gastrodin (**12**), 7-*O*-(4-hydroxybenzyl) gastrodin (**13**) and 6′-*O*-(4-hydroxybenzyl) gastrodin (**14**). Two phenolic glucosides (**1**, **2**) and two rare phenolic nucleosides (**5**, **11**) were preliminarily evaluated on bioactivity against glutamate-induced excitotoxicity by cell viability measurement and morphology. The cell viability values were evaluated by measuring the absorbance values of the CCK-8 reagent. Compounds **5** and **11** exhibited significant cytoprotective effects (*p* < 0.001), which may be related to the bioactivity of protecting the central nervous system.

## 2. Results

### 2.1. Elucidation of Chemical Structure

The purity of the six new compounds (**1**–**6**) was estimated to be over 90% by UHPLC analysis. Their chemical structures were searched on the SciFinder platform (https://scifinder-n.cas.org/, accessed on 21 April 2026). None of the six compounds has been previously reported or synthesized. Their key MS^2^ fragments, ^1^H-^1^H COSY and HMBC signals are shown in Figure 2. Their UV (Figure S1), IR (Figures S2–S7), HR-MS (Figures S9 and S10), UHPLC (Figures S11–S16) and NMR (Figures S17–S48) spectra are provided in the Supplementary Materials.

Compound **1** was isolated as a white powder. Based on the HR-ESI-MS data (found *m*/*z* 497.1816 [M-H]^−^, calculated for [C_27_H_29_O_9_]^−^, *m*/*z* 497.1800), compound **1** was determined as an isomer of 6′,7-di-*O*-(4-hydroxybenzyl) gastrodin, a known compound with the molecular formula C_27_H_30_O_9_, indicating thirteen degrees of unsaturation. In the MS^2^ spectra, the MS^2^ fragment at *m*/*z* 229.0849 ([C_14_H_13_O_3_]^−^) was derived from the precursor ion by sequential loss of a hydroxybenzyl and an anhydroglucose unit, indicating two hydroxybenzyl units linked by an ether bond. The ^1^H and ^13^C-NMR spectra contained the characteristic signals of a glucopyranosyl (Glc) and three 4-hydroxybenzyl moieties (designated as Bn1–Bn3), which was similar to 6′,7-di-*O*-(4-hydroxybenzyl) gastrodin [21]. Based on the 2D-NMR spectra, the ^1^H and ^13^C resonances were respectively assigned to the one Glc and three benzyl groups. The coupling constants (*J* = 7.6 Hz) between the protons at the C-1 and C-2 positions in the Glc group of the gastrodin moiety indicating the Glc group adopted the *β* configuration. In the HMBC spectrum, the 1-methine proton of the *β*-Glc group [*δ*_H_ 4.91 (d, 1H)] was correlated to a quaternary carbon of Bn1 (*δ*_C_ 158.78). This indicated that the 1-hydroxy proton of the *β*-Glc group was substituted by the quaternary carbon at the 4-position of the Bn1, forming the structure of gastrodin. The ^1^H and ^13^C signals of 7-methylene groups of Bn2 [*δ*_C_ 72.62, *δ*_H_ 4.43 (s, 2H)] and Bn1 [*δ*_C_ 72.44, *δ*_H_ 4.45 (s, 2H)] were correlated to each other. This indicated that the 7-methylene of Bn2 was linked to the 7-methylene in the gastrodin structure through an ether bond, which was consistent with 6′,7-bis-*O*-(4-hydroxybenzyl) gastrodin. Their difference was that the 7-methylene protons of Bn3 [*δ*_H_ 4.94 (s, 2H)] showed long-range correlation with the 4-carbon (*δ*_C_ 160.06) of Bn2, indicating that the hydrogen atom of the 4-hydroxyl group in Bn2 was substituted by Bn3. Therefore, compound **1** was elucidated as 7-[(4-hydroxybenzyloxy)-4-hydroxybenzyloxy] gastrodin and named gastrotribenzyloside A.

Compound **2** was isolated as a white powder. Based on the HR-ESI-MS data (found *m*/*z* 497.1770 [M-H]^−^, calculated for [C_27_H_29_O_9_]^−^, *m*/*z* 497.1806), compound **2** was determined as another isomer of 6′,7-di-*O*-(4-hydroxybenzyl) gastrodin with the molecular formula C_27_H_30_O_9_. The MS^2^ fragment at *m*/*z* 211.0737 ([C_14_H_11_O_2_]^−^) indicated the possibility of two directly linked hydroxybenzyl structures within the molecule. The ^1^H and ^13^C-NMR spectra of compound **2** closely matched the typical resonances of a Glc and another three benzyl groups (Bn1–Bn3), which was similar to compound **1**. Their difference was a special 3-(4-hydroxybenzyl)-4-hydroxybenzyl structure in compound **2**. The three aromatic protons with different chemical shifts [*δ*_H_ 6.97 (s, 1H), 6.72 (d, 8.2 Hz, 1H), and 6.98 (d, 8.2 Hz, 1H)] indicated a 3,4-substituted benzyl group. Based on 2D-NMR spectra, the signals were assigned to the 2-, 5- and 6-protons of Bn2. Similar to compound **1,** the quaternary carbon at the 4-position of Bn1 (*δ*_C_ 158.41) was attached to the 1-methine of the Glc group [*δ*_H_ 4.86 (m, 1H)] by an ether bond based on the HMBC spectrum, indicating a gastrodin moiety. The methylene protons of Bn-2 [*δ*_H_ 4.42 (d, 1H) and 4.39 (d, 1H)] were correlated to the 6-methylene carbon of the Glc group (*δ*_C_ 70.32) in the HMBC spectrum. This indicated that the 6-hydroxy proton was substituted by the 7-methylene of Bn-2. Moreover, the 7-methylene protons of Bn3 [*δ*_H_ 3.80 (d, 1H) and 3.77 (d, 1H)] was correlated to both C-3 and C-4 carbons of Bn2 [*δ*_C_ 155.91 (C-4) and 129.58 (C-3)]. It was suggested that the 7-methylene of Bn3 was connected to Bn2 at a quaternary carbon located no more than one bond away from both C-3 and C-4. The quaternary carbon C-4 was connected to a hydroxyl group. Therefore, the methylene group of Bn3 was attached to the C-3 position of Bn3, generating a 3-(4-hydroxybenzyl)-4-hydroxybenzyl moiety. For the absence of an electron-withdrawing oxygen atom, the chemical shifts of the methylene in Bn3 (*δ*_C_ 35.73; *δ*_H_ 3.80, 3.77) were obviously lower than those of the methylene groups in Bn1 and Bn2. Therefore, compound **2** was elucidated as 6′-*O*-[3-(4-hydroxybenzyl)-4-hydroxybenzyl] gastrodin and named gastrotribenzyloside B.

Compound **3** was isolated as a white powder. Based on the HR-ESI-MS data (found *m*/*z* 497.1792 [M-H]^−^, calculated for [C_27_H_29_O_9_]^−^, *m*/*z* 497.1806), it was determined as another isomer of 6′,7-di-*O*-(4-hydroxybenzyl) gastrodin with the molecular formula C_27_H_30_O_9_. The ^1^H and ^13^C-NMR spectra of compound **3** exhibited resonances of a Glc and three 4-substituted benzyl moieties (Bn1–Bn3), which were similar to compound **1**. In the HMBC spectrum, the correlation between the 1-methine proton of the Glc group [*δ*_H_ 4.87 (m, 1H)] and the C-4 quaternary carbon of Bn1 (*δ*_C_ 158.54) indicated a gastrodin structure, too. However, the other long-range correlations revealed the differences between compound **1** and **3** in the linkage positions of these structural moieties. Specifically, the 7-methylene protons [*δ*_H_ 4.44 (d, 1H) and 4.37 (d, 1H)] were correlated to the Glc-C-6 carbon (*δ*_C_ 69.73) in the HMBC spectra. It was suggested that the hydroxyl proton at the Glc-C-6 position was substituted by a 4-hydroxybenzyl moiety, which was similar to 6′,7-di-*O*-(4-hydroxybenzyl) gastrodin reported in Tianma [21]. Moreover, the C-4 methine proton of the Glc group [*δ*_H_ 3.43 (m, 1H)] was correlated to the 7-methylene carbon of Bn2 (*δ*_C_ 75.83) in the HMBC spectrum. It was indicated the hydroxyl proton at the Glc-C-4 position was substituted by another 4-hydroxybenzyl moiety. Therefore, compound **3** was elucidated as 4′,6′-di-*O*-(4-hydroxybzenzyl) gastrodin and named gastrotribenzyloside C.

Compound **4** was isolated as a white powder. Based on the HR-ESI-MS data (found *m*/*z* 603.2194 [M-H]^−^, calculated for [C_34_H_35_O_10_]^−^, *m*/*z* 603.2225), it was determined as an isomer of 6′,7-di-*O*-(4-hydroxybenzyl) gastrodin with the molecular formula C_27_H_30_O_9_, indicating seventeen degrees of unsaturation. The ^1^H and ^13^C NMR exhibited the characteristic signals of a glucopyranosyl group (Glc), two 4-substituted benzyl moieties (Bn1, Bn4) and two 3,4-substituted benzyl moieties (Bn2, Bn3). Similar to compound **2**, two groups of aromatic protons [*δ*_H_ 6.91 (d, 1H), 6.70 (d, 1H), and 6.97 (m, 1H) in Bn2; *δ*_H_ 6.83 (s, 1H), 6.64 (d, 1H), and 6.82 (d, 1H) in Bn3] were assigned to two 3,4-substituted benzyl moieties. The two methylene groups with lower chemical shifts [*δ*_C_ 35.72, *δ*_H_ 3.74 (d, 1H), and 3.71 (d, 1H); *δ*_C_ 35.78; 3.76 (s, 2H)] than other methylene signals indicated the absence of electron-withdrawing oxygen atoms. In the HMBC spectrum, the correlation between the Glc-1-methine proton [*δ*_H_ 4.86 (m, 1H)] and the C-4 quaternary carbon of Bn1 (*δ*_C_ 158.37) indicated a gastrodin structure. The correlation between the methylene protons of Bn2 [*δ*_H_ 4.40 (d, 1H) and 4.37 (d, 1H)] and the Glc-C-6 carbon (*δ*_C_ 70.25) indicated that the hydroxyl proton at the Glc-C-6 position was substituted by the methylene of Bn2. This structure was similar to compound **3**. Their difference was the positions of Bn3 and Bn4. In the HMBC spectrum, the methylene protons of Bn3 [*δ*_H_ 3.74 (d, 1H) and 3.71 (d, 1H)] were correlated to the aromatic carbons of Bn2 [*δ*_C_ 155.82 (C-4) and 128.60, (C-2)], which was similar to compound **2**. It was indicated that the 7-methylene of Bn3 must be connected to Bn2 at a quaternary carbon located no more than one bond away from both C-2 and C-4. Therefore, the methylene group of Bn3 was connected to the C-3 position of Bn2. Similarly, the methylene proton of Bn4 [*δ*_H_ 3.76 (s, 2H)] was correlated to the aromatic carbons of Bn3 [*δ*_C_ 154.01 (C-4) and 132.09 (C-2)], indicating that the methylene of Bn4 was connected to Bn3-C-3, too. Therefore, compound **4** was elucidated as 6′-*O*-{3-[3-(4-hydroxybenzyl)-4-hydroxybenzyl]-4-hydroxybenzyl} gastrodin and named gastrotetrabenzyloside D.

Compound **5** was isolated as a white powder. Its molecular formula was determined as C_24_H_25_N_5_O_6_ based on the HR-ESI-MS data (found *m*/*z* 478.1730 [M-H]^−^, calculated for [C_24_H_24_N_5_O_6_]^−^, *m*/*z* 478.1721), indicating fifteen degrees of unsaturation. The MS^2^ spectrum exhibited continuous neutral loss of 106.0339 Da (C_7_H_6_O) and 132.0404 Da (C_5_H_8_O_4_), which corresponded to the loss of dehydrated hydroxybenzyl and ribosyl groups. In the ^1^H and ^13^C-NMR spectra, the signals of adenine, ribosyl and 4-hydroxybenzyl groups matched closely with *N*^6^-(4-hydroxybenzyl) adenosine reported in Tianma [22]. In the HMBC spectrum, the 1-methine proton of the ribosyl group [*δ*_H_ 5.98 (d, 1H)] was correlated to the carbons in the adenine moiety (*δ*_C_ 151.20, C-4; *δ*_C_ 140.26, C-8). It was indicated that the 9-amino proton was substituted by the ribosyl group supporting the adenosine structure. Their difference was that two chemically equivalent 4-hydroxybenzyl groups in compound **5** shared identical resonances. This observation indicated the substitution of the 4-hydroxybenzyl groups occurred at the only possible position of the 6-amino group in the adenine moiety. In the HMBC spectrum, the correlation between the 7-methylene proton of the hydroxybenzyl groups [*δ*_H_ 3.82 (m, 4H)] and the C-6 carbon in the adenine moiety (*δ*_C_ 156.19) verified that the two 4-hydroxybenzyl groups were connected at the 6-amino group in the adenine moiety. Moreover, the ^1^H and ^13^C-NMR signals of both the 7-methylenes of the hydroxybenzyl groups displayed very weak intensity. This weak intensity might be ascribed to the unstable conformations of compound **5** caused by the delocalization of the lone pair electrons from the 6-amino group in the adenine moiety, leading to the reversible formation of a carbon–nitrogen double bond. Therefore, compound **5** was elucidated as *N*^6^-di-(4-hydroxybenzyl) adenosine and named gastronucleoside B.

Compound **6** was isolated as a white powder. Its molecular formula was determined as C_31_H_31_N_5_O_7_ based on the HR-ESI-MS data (found *m*/*z* 584.2117 [M-H]^−^, calculated for [C_31_H_30_N_5_O_7_]^−^, *m*/*z* 584.2140), indicating nineteen degrees of unsaturation. The MS^2^ fragments at *m*/*z* 478.1674, 372.1292 and the characteristic fragment of an adenosine at *m*/*z* 266.0875 ([C_10_H_12_N_5_O_4_]^−^) were derived from the precursor ion by sequential neutral loss of three hydroxybenzyl groups. This indicated that the molecular structure contained one more hydroxybenzyl moiety than compound **5**. The ^1^H and ^13^C-NMR spectra of compound **6** were similar to compound **5** and exhibited signals of one more hydroxybenzyl moiety compared to compound **5**, which confirmed this inference. Moreover, three aromatic protons with different chemical shifts [*δ*_H_ 6.92 (dd, 1H), 6.79 (d, 1H), and 6.71 (d, 1H)] indicated a 3,4-substituted benzyl group. The ^1^H and ^13^C resonances were respectively assigned to one 3,4-substituted benzyl (Bn1), two 4-substituted benzyls (Bn2 and Bn3) and one adenosine structure. In the HMBC spectrum, the correlation from the 7-methylene protons [*δ*_H_ 3.83–3.77 (m, 4H)] of Bn1 and Bn3 to the C-6 carbon in the adenine moiety (*δ*_C_ 156.05) indicated that the two protons at the 6-amino group of the adenine were replaced by 7-methylenes of Bn1 and Bn3. Similar to compound **5**, the ^1^H and ^13^C-NMR signals of 7-methylenes in Bn1 and Bn3 [*δ*_C_ 35.63; 3.83–3.77 (m, 2H)] displayed very weak intensity, which might be caused by the delocalization of the lone pair electrons from the 6-amino group in the adenine moiety. Similar to compounds **2** and **4**, the methylene protons of Bn2 [*δ*_H_ 3.77 (s, 2H)] were correlated to the C-4 quaternary carbon of Bn1 (*δ*_C_ 155.40) in the HMBC spectrum. It was indicated that the only possible connecting position of the 7-methylene group of Bn2 was the C-3 position of Bn1. Therefore, compound **6** was elucidated as *N*^6^-(4-hydroxybenzyl)-*N*^6^-[3-(4-hydroxybenzyl)-4-hydroxybenzyl] adenosine and named gastronucleoside C.

Along with the six new compounds, eight known compounds were identified by analyzing their spectroscopic data and comparing them with relevant studies. These compounds were 2-[4-hydroxy-3-(4-hydroxybenzyl) benzyl]-4-methoxymethyl phenol (**7**), 6′,7-di-*O*-(4-hydroxybenzyl) gastrodin (**8**) [21], 4-hydroxy-3-(4-hydroxybenzyl) benzyl methyl ether (**9**) [23], 2′,7-di-*O*-(4-hydroxybenzyl) gastrodin (**10**) [21], *N*^6^-(4-hydroxybenzyl) adenosine (**11**) [22], 2′-*O*-(4-hydroxybenzyl) gastrodin (**12**), 7-*O*-(4-hydroxybenzyl) gastrodin (**13**), and 6′-*O*-(4-hydroxybenzyl) gastrodin (**14**) [21].

The spectroscopic data of the six new compounds are presented as follows:

Gastrotribenzyloside A (**1**): UV (CH_3_OH) *λ*_max_: 192, 222, 274 nm. IR (KBr) *ν*_max_ (cm^−1^): 3390, 2863, 1613, 1513, 1448, 1364, 1233, 1172, 1074, 1017, 828 cm^−1^. HR-ESI-MS (*m*/*z*): 497.1800 [M-H]^−^ (calculated for [C_27_H_29_O_9_]^−^, *m*/*z* 497.1806). NMR (DMSO-*d*_6_) data are shown in Table 1.

Gastrotribenzyloside B (**2**): UV (CH_3_OH) *λ*_max_: 192, 224, 277 nm. IR (KBr) *ν*_max_ (cm^−1^): 3420, 2918, 2873, 1613, 1511, 1440, 1363, 1231, 1061, 1012, 825, 778 cm^−1^. HR-ESI-MS (*m*/*z*): 497.1770 [M-H]^−^ (calculated for [C_27_H_29_O_9_]^−^, *m*/*z* 497.1806). NMR (CD_3_OD) data are shown in Table 1.

Gastrotribenzyloside C (**3**): UV (CH_3_OH) *λ*_max_: 192, 222, 273 nm. IR (KBr) *ν*_max_ (cm^−1^): 3420, 2917, 2876, 1613, 1512, 1439, 1363, 1259, 1233, 1058, 1025, 1000, 826, 769 cm^−1^. HR-ESI-MS (*m*/*z*): 497.1792 [M-H]^−^ (calculated for [C_27_H_29_O_9_]^−^, *m*/*z* 497.1806). NMR (CD_3_OD) data are shown in Table 1.

Gastrotetrabenzyloside D (**4**): UV (CH_3_OH) *λ*_max_: 192, 227, 273 nm. IR (KBr) *ν*_max_ (cm^−1^): 3418, 2915, 2874, 1612, 1514, 1450, 1365, 1231, 1060, 827 cm^−1^. HR-ESI-MS (*m*/*z*): 603.2194 [M-H]^−^ (calculated for [C_34_H_35_O_10_]^−^, *m*/*z* 603.2225). NMR (CD_3_OD) data are shown in Table 2.

Gastronucleoside B (**5**): UV (CH_3_OH) *λ*_max_: 191, 281 nm. IR (KBr) *ν*_max_ (cm^−1^): 3407, 2926, 1667, 1588, 1514, 1445, 1346, 1232, 1172, 1078, 1048, 987, 826 cm^−1^. HR-ESI-MS (*m*/*z*): 478.1730 [M-H]^−^ (calculated for [C_24_H_24_N_5_O_6_]^−^, *m*/*z* 478.1721). NMR (CD_3_OD) data are shown in Table 3.

Gastronucleoside C (**6**): UV (CH_3_OH) *λ*_max_: 191, 281 nm. IR (KBr) *ν*_max_ (cm^−1^): 3418, 2924, 1587, 1512, 1440, 1344, 1230, 1175, 1107, 1076, 1047, 987, 900, 825 cm^−1^. HR-ESI-MS (*m*/*z*): 584.2117 [M-H]^−^ ([C_31_H_30_N_5_O_7_]^−^, *m*/*z* 584.2140). NMR (CD_3_OD) data are shown in Table 4.

### 2.2. Screening of Bioactivity Against Glutamate-Induced Excitotoxicity

**Figure 3:**
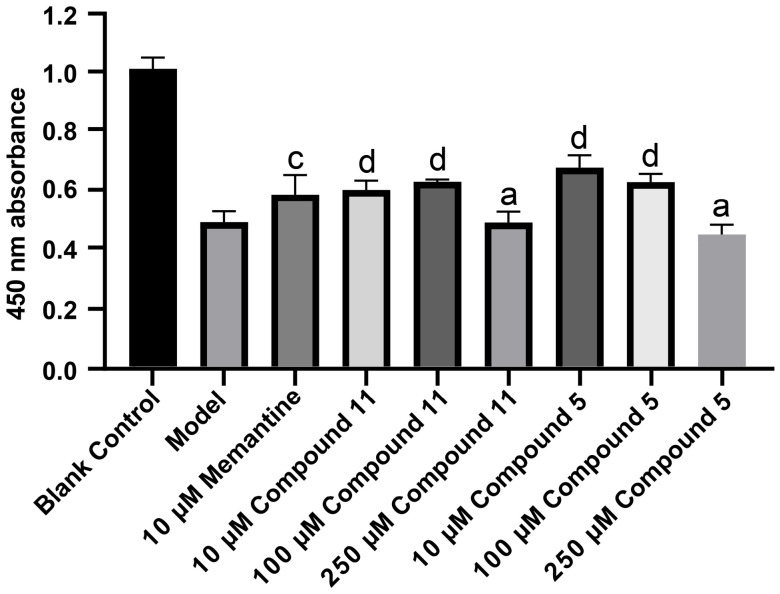
Cell viability of compounds **5** and **11**. Statistical comparisons were performed by one-way analysis of variance with Dunnett’s test. The results are shown as the mean ± standard deviation (*n* = 5). a. *p* ≥ 0.05; c. *p* < 0.01; d. *p* < 0.001, compared to the model group.

**Figure 4:**
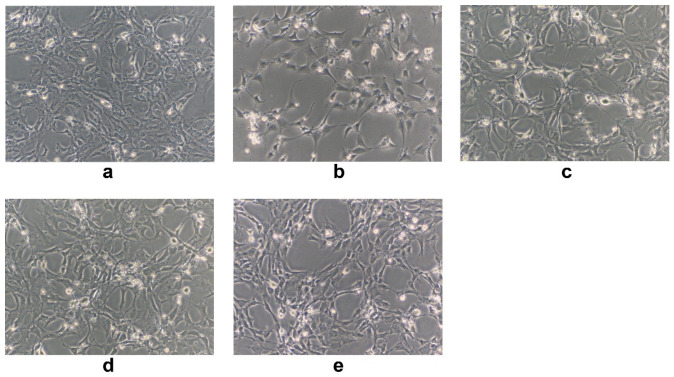
Cell morphology of compounds **5** and **11**. (**a**) Blank control group; (**b**) glutamate-treated model group; (**c**) 10 μM memantine-treated group; (**d**) 10 μM compound **11**-treated group; (**e**) 10 μM compound **5**-treated group.

**Figure 5:**
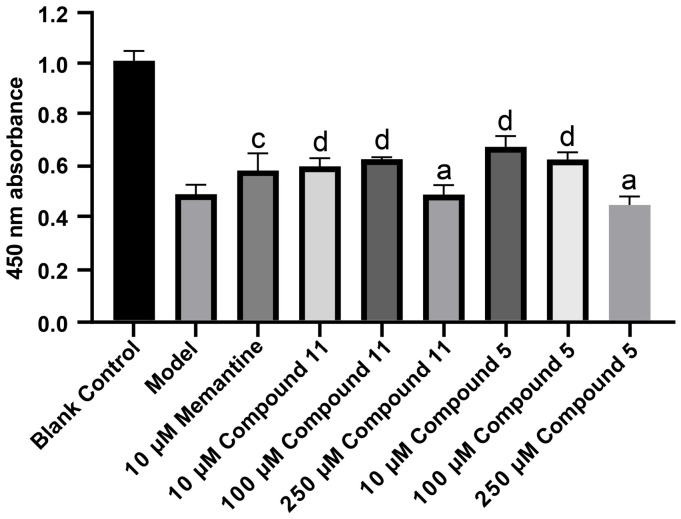
Cell viability of compounds **1** and **2**. Statistical comparisons were performed by one-way analysis of variance with Dunnett’s test. The results are shown as the mean ± standard deviation (*n* = 5). a. *p* ≥ 0.05; c. *p* < 0.01; d. *p* < 0.001; compared to the model group.

A cell viability assay showed that treatment with 15 mM glutamate reduced cell viability by 51.5% compared with the blank control group. Compounds **5** and **11** exhibited very significant effect (*p* < 0.001) on the increase in cell viability reduced by glutamate at both the low and medium concentrations (Figure 3). In contrast to the glutamate-treated model group, the cell viability values of groups treated with 10 μM and 100 μM compound **11** increased by 10.8% and 13.6%, respectively. Those treated with 10 μM and 100 μM compound **5** increased by 18.3% and 13.5%, respectively. Compounds **5** and **11** at 250 μM concentration did not show improving effects. The morphological changes also indicated that compounds **5** and **11** enhanced cell viability under glutamate-induced excitotoxicity (Figure 4). However, two phenolic glucosides, compounds **1** and **2**, did not increase the cell viability reduced by glutamate (*p* > 0.05) at any of the three concentrations (Figure 5). The original data of cell viability are shown in Tables S1 and S2 of the Supplementary Materials.

## 3. Discussion

### 3.1. Elucidation of Structure Units by MS^2^ Fragments

In this study, six new compounds were elucidated primarily by combining HR-ESI-MS/MS and HMR analysis. Firstly, the main structure units were inferred from characteristic MS_2_ fragments or neutral loss, which was based on our earlier MS/MS study on known compounds in Tianma (Figure S8 in the Supplementary Materials). The common structure units included: gastrodin *m*/*z* 285.0974, neutral loss of 286.1053 or 268.0947; 4-hydroxybenzyl, *m*/*z* 123.0446, neutral loss of 124.0524 or 106.0419; glucopyranosyl, *m*/*z* 161.0450 or 179.0556, neutral loss of 162.0528; 3-(4-hydroxybenzyl)-4-hydroxybenzyl, *m*/*z* 211.0759; ribosyl, neutral loss 132.0423. Then the structure units inferred by MS^2^ spectra could be confirmed or corrected by ^1^H, ^13^C, DEPT and even 2D-NMR spectra. Finally, the connecting positions of the units were confirmed by HMBC spectra.

### 3.2. Determination of Special 3-(4-Hydroxybenzyl)-4-Hydroxybenzyl Moiety in Compounds **2**, **4** and **6**

The MS^2^ and NMR spectra of compounds **1**–**6** showed characteristic signals including multiple hydroxybenzyl groups. The point that needs to be emphasized was the elucidation of the special structure unit of 3-(4-hydroxybenzyl)-4-hydroxybenzyl of compounds **2**, **4** and **6**, which was different from compounds **1**, **3** and **5**. However, the characteristic fragment at 211.0737 was detected exclusively in the MS^2^ spectrum of only **2**. In the aromatic regions of ^1^H spectra, compounds **1**, **3** and **5** showed the typical resonances integrating to 2H of protons in 4-substituted benzyl groups, which could be confirmed by their ortho coupling in the ^1^H-^1^H COSY spectra. Moreover, the ^1^H and ^13^C chemical shifts of 7-methylenes (*δ*_H_ 4.3–5.0; *δ*_C_ 64–76) and 4-quaternary carbons (*δ*_C_ 155–160) in the benzyl groups indicated that they were connected to an electron-withdrawing ether bond or hydroxyl group.

In contrast, the compounds **2**, **4** and **6** exhibited signals of three different protons in the aromatic regions. Two of them were correlated to each other in the ^1^H-^1^H COSY spectra. Based on their correlation to the 7-methylene carbon of the benzyl group in the HMBC spectra, the three protons could be confirmed as 2-, 5-, and 6-H in the benzyl group. Therefore, it could be indicated that compounds **2**, **4** and **6** contained 1,3,4-substituted benzyl groups. Moreover, some methylene groups exhibited lower ^1^H and ^13^C chemical shifts (*δ*_H_ 3.7–3.9; *δ*_C_ 35–36) compared to compounds **1** and **3**. In the HMBC spectra, the correlation between the 7-methylene protons and the C-2, 3 or 4 carbon of a second benzyl group could doubtlessly confirm that the methylene group was substituted at the C-3 quaternary carbon through a carbon–carbon single bond. Based on the information above, the special 3-(4-hydroxybenzyl)-4-hydroxybenzyl structure of compounds **2**, **4**, and **6** was confirmed.

### 3.3. Inference and Limitations of Bioactive Experiments

In the in vitro experiments, two phenolic glucosides (**1**, **2**) and two phenolic nucleosides (**5**, **11**) were preliminarily evaluated on cytoprotective effects against the glutamate-induced excitotoxicity. Recent studies considered that the effects of phenolic glucosides in Tianma were attributed to their in vivo hydrolysis to generate 4-hydroxybenzyl alcohol and gastrodin, which were important bioactive components in Tianma [24,25]. Compounds **1** and **2** did not show protective effects. It was inferred that phenolic glucosides **1** and **2** may not be effectively hydrolyzed under the in vitro cell culture conditions. Compounds **5** and **11** exhibited significant effects of increasing cell viability compared to the model group. It could be inferred that their bioactive mechanisms on the nervous system were different from phenolic glucosides. The results indicated the potential of phenolic nucleosides in Tianma for further pharmaceutical investigation.

This manuscript mainly focused on discovering new compounds and did not provide the whole direct experimental link between the isolated compounds and the biological activity in the ethyl acetate extract of Tianma. The mechanistic studies were not performed due to the limited quantities of the isolated compounds. The cytoprotective effect against excitotoxicity still remains to be verified by in vivo studies. Therefore, these results of the bioactive experiment should be considered hypothesis-generating. Further investigation is required to elucidate in vivo effects and the underlying mechanisms.

## 4. Materials and Methods

### 4.1. General

Column chromatography was carried out on silica gel (45–75 μm, Qingdao Marine Chemical Factory, Qingdao, China) and C_18_-bonded reversed silica gel (Welchrom C_18_^E^, 40–63 μm, Welch Technology, Shanghai, China). Thin-layer chromatography was performed on silica gel GF_254_ (Qingdao Marine Chemical Factory, Qingdao, China). Semi-preparative HPLC was conducted by a PuriMaster-3000A instrument with an Ultimate-XB C_18_ column (5 μm, 10 × 250 mm, Welch Technology, Shanghai, China). NMR spectra were recorded by an AVANCE NEO 600-NMR spectrometer (Bruker, Karlsruhe, Germany) with TMS as the internal standard. Mass spectra were acquired by an Impact II Q-TOF mass spectrometer (Bruker, Karlsruhe, Germany) equipped with an electrospray ionization source. IR spectra were recorded by a Cary 610 spectrometer (Agilent Technologies Inc., Santa Clara, CA, USA) with KBr pellets. The cell viabilities were measured by a MD SpectraMax iD5 microplate reader (Molecular Devices Shanghai Corporation, Shanghai, China).

### 4.2. Plant Materials

The Tianma sample was provided by Sichuan Chijian Traditional Chinese Medicine Technology Co., Ltd. (Chengdu, China). The sample was authenticated as tubers of *Gastrodia elata* Blume by Professor Guanghua Lu from the Chengdu University of Traditional Chinese Medicine. This specimen (No. 20211207) was stored in the herbarium of this institution.

### 4.3. Extraction and Isolation

The Tianma sample (7.5 kg) was refluxed in 90% ethanol three times (8:1, 7:1 and 6:1 *v*/*w*, 1 h each) to obtain ethanolic extract (358.1 g, A). Then the material residue was refluxed in water four times (14:1, 12:1, 10:1 and 10:1 *v*/*w*, 1 h each) to obtain aqueous extract (3212.0 g, B). Extract B was concentrated and then precipitated at a concentration of 80% ethanol. After filtration, the filtrate (C) was merged with extract A, and then concentrated under vacuum to yield the total extract (813.5 g, D). Extract D was suspended in 3000 mL water and then successively extracted with petroleum ether and ethyl acetate (1:1, *v*/*v*) three times to yield petroleum ether fraction (29.3 g), ethyl acetate fraction (90.5 g) and water fraction (689.8 g). The ethyl acetate extract was separated by silica gel column chromatography (45–75 μm, 9 × 60 cm) and eluted with CH_2_Cl_2_-CH_3_OH (19:1 to 0:1, *v*/*v*) to obtain seven crude fractions (Fr.1–Fr.7).

Fr.4 (9.3 g) was fractionated by reversed-phase C_18_ column chromatography (Welchrom C_18_^E^, 40–63 μm, 5.5 × 30 cm) and eluted with H_2_O-CH_3_OH (9:1 to 1:9, *v*/*v*) to yield fractions Fr.4.1–Fr.4.15. Fr.4.14 (0.40 g) was fractionated by reversed-phase C_18_ column chromatography and eluted with H_2_O-CH_3_OH (5:5 to 2:8, *v*/*v*) to yield fractions Fr.4.14.1–Fr.4.14.3. Fr.4.14.2 (170 mg) was separated by semi-preparative HPLC and eluted with H_2_O-CH_3_CN (65:35 to 60:40, *v*/*v*; 3 mL/min) to obtain compounds **1** (6.9 mg) and **7** (8.7 mg). Fr.4.10 (0.22 g) was fractionated by semi-preparative HPLC and eluted with H_2_O-CH_3_CN (64:36 to 36:28, *v*/*v*) to obtain fractions Fr.4.10.1–Fr.4.10.3. Then Fr.4.10.2 (30 mg) was separated by semi-preparative HPLC and eluted with H_2_O-CH_3_OH (50:50 to 47:53, *v*/*v*; 2.5 mL/min) to obtain compound **2** (6.3 mg). Fr.4.12 (0.19 g) was separated by semi-preparative HPLC and eluted with H_2_O-CH_3_OH (45:55 to 42:58, *v*/*v*; 2.5 mL/min) to obtain compound **8** (20.2 mg). The remaining eluate of Fr.4.12 was collected and concentrated under vacuum. Then the concentrated residue was separated by semi-preparative HPLC and eluted with H_2_O-CH_3_CN (65:35 *v*/*v*; 3 mL/min) to obtain compounds **4** (4.8 mg) and **9** (4.7 mg).

Fraction Fr.5 (5.8 g) was fractionated by reversed-phase C_18_ column chromatography and eluted with H_2_O-CH_3_OH (9:1 to 1:9, *v*/*v*) to yield fractions Fr.5.1–Fr.5.13. Fr.5.8 (0.25 g) was separated by semi-preparative HPLC and eluted with H_2_O-CH_3_CN (80:20 to 70:30, *v*/*v*; 3 mL/min) to obtain compounds **3** (4.5 mg) and **5** (28.0 mg). Fr.5.10 (0.33 g) was separated by semi-preparative HPLC, on a reversed-phase C_18_ column chromatography and eluted with H_2_O-CH_3_OH (5:5 to 2:8) to yield fractions Fr.5.10.1–Fr.5.10.4. Fr.5.10.2 (22 mg) was separated by semi-preparative HPLC and eluted with H_2_O-CH_3_CN (70:30 to 65:35, *v*/*v*; 3 mL/min) to obtain compound **6** (3.6 mg). Fr.5.10.1 (32 mg) was separated by the same method as Fr.5.10.2 to obtain compound **10** (4.1 mg). Fr.5.6 was separated by semi-preparative HPLC and eluted with H_2_O-CH_3_OH (74:26 to 72:28, *v*/*v*; 2.5 mL/min) to obtain compounds **11** (13.7 mg), **12** (26.8 mg) and **13** (29.8 mg). The remaining eluate of Fr.5.6 was collected and concentrated under vacuum. Then the concentrated residue was separated by semi-preparative HPLC and eluted with H_2_O-CH_3_CN (87:13 to 85:15, *v*/*v*; 3 mL/min) to obtain compound **14** (50.3 mg).

### 4.4. Structure Elucidation

Firstly, ^13^C-NMR data were compared with those in the online NMR database (http://www.nmrdata.com/, accessed on 15 February 2026) to identify known compounds. Then main chemical groups of unidentified were predicted based on the ^1^H and ^13^C-NMR spectra, DEPT 90 and 135 spectra, and MS^2^ fragments. Afterward, the correlations among the chemical groups were elucidated by ^1^H-^1^H COSY, HSQC and HMBC. Finally, the elucidated compounds were searched on SciFinder (https://scifinder-n.cas.org/, accessed on 21 April 2026) to confirm if they were unreported.

### 4.5. Cell Culture

HT-22 cells were cultured in 10% fetal bovine serum (Gibco, Thermo Fisher Scientific, Waltham, MA, USA) and 1% penicillin/streptomycin solution with 5% CO_2_ in DMEM supplemented at 37 °C. HT-22 cells were used between passages 8 and 12.

### 4.6. Cell Viability Assay

HT-22 cells (1 × 10^4^ cells/well) were plated overnight in 96-well plates. Compounds **5** and **11** were dissolved in serum-free DMEM without co-solvent to prepare 2 mM stock solutions, which were then sterilized by filtration through a 0.22 μm membrane filter. After washing with serum-free PBS, the cells were pretreated with 10, 100, and 250 μM compounds **1**, **2**, **5** and **11** or 10 μM memantine for 24 h, respectively. Subsequently, they were treated with 15 mM glutamic acid for 36 h. Then 10 μL of CCK-8 reagent was added to each well, followed by incubation for 2 h at 37 °C in a 5% CO_2_ atmosphere [26,27]. Absorbance was measured at 450 nm. Cell viability was calculated relative to untreated controls, which were set at 100%. Experiments were performed in pentaplicate (*n* = 5). Data were presented as mean ± standard deviation. All statistical analyses were conducted using GraphPad Prism (version 8.2.1). Statistical comparisons were performed using one-way analysis of variance with Dunnett’s test. A value of *p* < 0.05 was considered statistically significant.

HT-22 cells (3 × 10^5^ cells/well) were plated overnight in 6-well plates and pretreated with 10 μM compounds **5** and **11**. Subsequently, they were treated with 15 mM glutamic acid for 36 h. Cell morphology was then observed by an inverted microscope.

## 5. Conclusions

Tianma is a well-known medicinal and edible herb. There are many compounds remaining undiscovered in Tianma, which hinders further pharmacological research and product development. In order to obtain more chemical information on Tianma, the chemical constituents are investigated in this study, resulting in 14 compounds isolated and identified. These compounds comprise nine phenolic glucosides (**1**–**4**, **8**, **10** and **12**–**14**), three phenolic nucleosides (**5**, **6** and **11**) and two polyphenols (**7** and **9**). Among them, compounds **1**–**6** are new natural compounds, which provide new chemical information on Tianma. Compounds **5** and **6** are the first reported phenolic nucleosides substituted by multiple benzyl groups in Tianma. Furthermore, two phenolic glucosides (**1** and **2**) and two phenolic nucleosides (**5** and **11**) are preliminarily evaluated for bioactivity. Compounds **5** and **11** exhibit protective effects by increasing the cell viability reduced by glutamate-induced excitotoxicity in vitro, while compounds **1** and **2** do not exhibit cytoprotective effects. These results indicate that phenolic nucleosides may also be potential bioactive components in Tianma, which remains to be verified in further investigation.

These findings not only enrich the chemical diversity of Tianma but also suggest that compounds of rare categories may represent a potential class of neuroprotection worthy of further investigation. However, it should be noted that the current bioactivity screening is limited to an in vitro HT-22 cell model without extrapolating to human disease. The underlying mechanisms as well as in vivo efficacy remain to be clarified. Moreover, the whole link between the isolated compounds and the biological activity in ethyl acetate extract of Tianma has not been revealed. Future studies will focus on the targeted isolation of additional phenolic nucleoside analogues, the elucidation of their bioactive mechanisms, and the validation of their bioactivity in animal models.

## Figures and Tables

**Figure 2:**
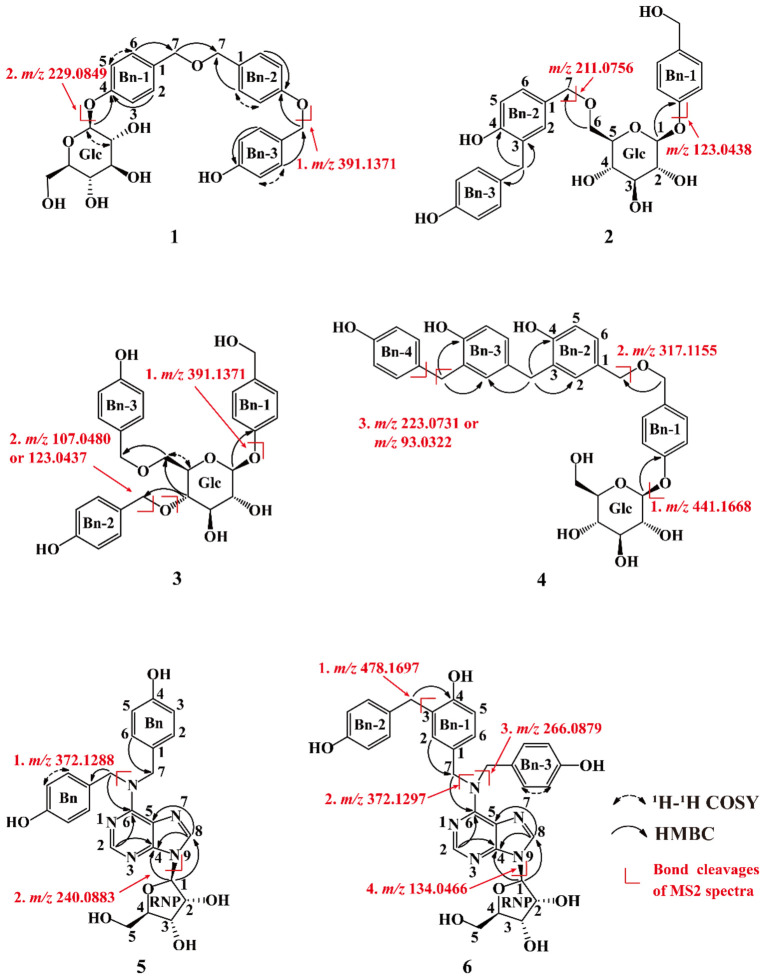
Characteristic MS^2^ fragments and 2D-NMR correlations of six new compounds: (**1**) gastrotribenzyloside A; (**2**) gastrotribenzyloside B; (**3**) gastrotribenzyloside C; (**4**) gastrotetrabenzyloside D; (**5**) gastronucleoside B; (**6**) gastronucleoside C.

**Table 1:** ^1^H and ^13^C-NMR data of compounds **1**–**3** (600 MHz for ^1^H, 150 MHz for ^13^C, *δ* in ppm, *J* in Hz, CD_3_OD).

Position	Compound 1		Compound 2		Compound 3	
	*δ*_C_	*δ*_H_	*δ*_C_	*δ*_H_	*δ*_C_	*δ*_H_
Bn1-1	133.40	-	136.62	-	136.68	-
Bn1-2,6	130.65	7.27 (d, 8.7 Hz, 2H)	129.46	7.24 (d, 8.5 Hz, 2H)	129.43	7.26 (d, 8.6, 2H)
Bn1-3,5	117.64	7.08 (d, 8.7 Hz, 2H)	117.72	7.07 (d, 8.5 Hz, 2H)	117.72	7.06 (d, 8.6, 2H)
Bn1-4	158.78	-	158.41	-	158.39	-
Bn1-7	72.44	4.45 (s, 2H)	64.85	4.52 (s, 2H)	64.84	4.54 (s, 2H)
Bn2-1	131.64	-	130.19	-	130.62	-
Bn2-2,6	130.65	7.24 (d, 8.5 Hz, 2H)	131.65 (C-2) 128.31 (C-6)	6.97 (s, 1H) 6.98 (d, 8.2 Hz, 1H)	131.17	7.09 (d, 8.4, 2H)
Bn2-3,5	115.87	6.95 (d, 8.6 Hz, 2H)	129.58 (C-3) 115.79 (C-5)	- 6.72 (d, 8.2 Hz, 1H)	116.11	6.73 (d, 8.4, 2H)
Bn2-4	160.06	-	155.91	-	158.32	-
Bn2-7	72.62	4.43 (s, 2H)	74.45	4.42 (d, 11.3 Hz, 1H) 4.39 (d, 11.3 Hz, 1H)	75.68	4.78 (d, 10.5 Hz, 1H) 4.44 (d, 10.5 Hz 1H)
Bn3-1	129.37	-	133.51	-	130.13	-
Bn3-2,6	130.49	7.24 (d, 8.5 Hz, 2H)	130.83	7.00 (d, 8.4 Hz, 2H)	130.99	7.14 (d, 8.4, 2H)
Bn3-3,5	116.20	6.78 (d, 8.6 Hz, 2H)	115.96	6.65 (d, 8.4 Hz, 2H)	116.03	6.73 (d, 8.4, 2H)
Bn3-4	158.40	-	156.28	-	158.31	-
Bn3-7	71.05	4.94 (s, 2H)	35.73	3.80 (d, 14.9 Hz, 1H) 3.77 (d, 14.9 Hz, 1H)	74.16	4.44 (d, 11.5 Hz, 1H) 4.37 (d, 11.5 Hz, 1H)
Glc-1	102.30	4.91 (d, 7.6 Hz, 1H)	102.28	4.86 (d, 7.5, 1H)	102.22	4.87 (m, 1H)
Glc-2	77.97	3.46 (m, 1H)	77.99	3.44 (m, 1H)	75.15	3.47 (dd, 9.2, 7.9 Hz, 1H)
Glc-3	74.91	3.47 (m, 1H)	71.77	3.33 (m, 1H)	76.07	3.57 (m, 1H)
Glc-4	71.36	3.40 (m, 1H)	74.89	3.45 (m, 1H)	78.75	3.43 (m, 1H)
Glc-5	78.12	3.44 (ddd, 9.8, 5.5, 2.1 Hz, 1H)	77.12	3.56 (m, 1H)	78.42	3.61 (m, 1H)
Glc-6	62.49	3.89 (dd, 12.1, 2.1 Hz, 1H) 3.70 (dd, 12.1, 5.5 Hz, 1H)	70.32	3.82 (d, 9.0 Hz, 1H) 3.58 (m, 1H)	69.58	3.68 (m, 1H) 3.57 (m, 1H)

**Table 2:** ^1^H and ^13^C-NMR data of compound **4** (600 MHz for ^1^H, 150 MHz for ^13^C, *δ* in ppm, *J* in Hz, CD_3_OD).

Position	*δ*_C_	*δ*_H_	Position	*δ*_C_	*δ*_H_
Bn1-1	136.60	-	Bn3-4	154.01	-
Bn1-2,6	129.49	7.22 (d, 8.4 Hz, 2H)	Bn3-5	115.80	6.64 (d, 8.2, 1H)
Bn1-3,5	117.73	7.05 (d, 8.4 Hz, 2H)	Bn3-6	128.42	6.82 (d, 8.2, 1H)
Bn1-4	158.37	-	Bn3-7	35.72	3.74 (d, 14.9 Hz, 1H) 3.71 (d, 14.9 Hz, 1H)
Bn1-7	64.84	4.50 (s, 2H)	Bn4-1	133.77	-
Bn2-1	130.13	-	Bn4-2,6	130.81	6.98 (d, 8.2 Hz, 2H)
Bn2-2	131.65	6.91 (d, 2.1 Hz, 1H)	Bn4-3,5	115.93	6.65 (d, 8.2 Hz, 2H)
Bn2-3	129.60	-	Bn4-4	156.12	-
Bn2-4	155.82	-	Bn4-7	35.78	3.76 (s, 2H)
Bn2-5	115.86	6.70 (d, 8.1 Hz, 1H)	Glc-1	102.25	4.86 (m, 1H)
Bn2-6	128.24	6.97 (m, 1H)	Glc-2	74.88	3.45 (m, 1H)
Bn2-7	74.46	4.40 (d, 11.2, 1H) 4.37 (d, 11.2, 1H)	Glc-3	77.10	3.45 (m, 1H)
Bn3-1	133.34	-	Glc-4	71.76	3.35 (m, 1H)
Bn3-2	132.09	6.83 (s, 1H)	Glc-5	77.97	3.56 (m, 1H)
Bn3-3	129.60	-	Glc-6	70.25	3.81 (dd, 12.1, 4.0, 1H) 3.57 (dd, 12.1, 6.4, 1H)

**Table 3:** ^1^H and ^13^C-NMR data of compound **5** (600 MHz for ^1^H, 150 MHz for ^13^C, *δ* in ppm, *J* in Hz, CD_3_OD).

Position	*δ*_C_	*δ*_H_	Position	*δ*_C_	*δ*_H_
2 × Bn-1	129.57	-	Adenine-6	156.19	-
2 × Bn-2,6	130.35	7.07 (d, 8.5 Hz, 4H)	Adenine-8	140.26	8.18 (s, 1H)
2 × Bn-3,5	116.32	6.72 (d, 8.5 Hz, 4H)	RNP-1	91.23	5.98 (d, 6.5 Hz, 1H)
2 × Bn-4	157.87	-	RNP-2	75.22	4.80 (dd, 6.5, 5.1 Hz, 1H)
2 × Bn-7	35.69	3.82 (m, 4H)	RNP-3	72.75	4.34 (dd, 5.1, 2.5 Hz, 1H)
Adenine-2	152.90	8.26 (s, 1H)	RNP-4	88.17	4.19 (m, 1H)
Adenine-4	151.20	-	RNP-5	63.57	3.90 (dd, 12.6, 2.4 Hz, 1H) 3.75 (dd, 12.6, 2.7 Hz, 1H)
Adenine-5	121.59	-			

**Table 4:** ^1^H and ^13^C-NMR data of compound **6** (600 MHz for ^1^H, 150 MHz for ^13^C, *δ* in ppm, *J* in Hz, CD_3_OD).

Position	*δ*_C_	*δ*_H_	Position	*δ*_C_	*δ*_H_
Bn1-1	129.96	-	Bn3-3,5	116.03	6.66 (d, 8.4 Hz, 2H)
Bn1-2	131.42	6.79 (d, 2.2 Hz, 1H)	Bn3-4	156.25	-
Bn1-3	129.56	-	Bn3-7	35.63	3.83–3.77 (m, 2H)
Bn1-4	155.40	-	Adenine-2	152.85	8.22 (s, 1H)
Bn1-5	115.96	6.70 (d, 8.1 Hz, 1H)	Adenine-4	151.12	-
Bn1-6	127.82	6.92 (dd, 8.1, 2.2 Hz, 1H)	Adenine-5	121.47	-
Bn1-7	35.63	3.83–3.77 (m, 2H)	Adenine-6	156.05	-
Bn2-1	129.31	-	Adenine-8	140.19	8.16 (s, 1H)
Bn2-2,6	130.42	6.99 (d, 8.5 Hz, 2H)	RNP-1	91.15	5.99 (d, 6.5 Hz, 1H)
Bn2-3,5	116.33	6.69 (d, 8.5 Hz, 2H)	RNP-2	75.21	4.81 (dd, 6.3, 5.2,1H)
Bn2-4	157.73	-	RNP-3	72.72	4.34 (dd, 5.2, 2.5 Hz, 1H)
Bn2-7	35.47	3.77 (s, 2H)	RNP-4	88.14	4.20 (m, 1H)
Bn3-1	133.19	-	RNP-5	63.54	3.91 (dd, 12.6, 2.4 Hz, 1H) 3.76 (dd, 12.6, 2.6 Hz, 1H)
Bn3-2,6	131.04	6.95 (d, 8.4 Hz, 2H)			

## Data Availability

Data is contained within the article.

## Supplementary Materials

The following supporting information can be downloaded at: https://www.mdpi.com/article/10.3390/ph19071068/s1, Figure S1: UV spectra of six new compounds isolated from Tianma; Figures S2–S7: FT-IR spectrum of compound **1**–**6**; Figure S8: MS^2^ fragments and neutral losses of typical compounds reported in Tianma; Figure S9: HRMS spectra of six new compounds isolated from Tianma; Figure S10: MS^2^ spectra of six new compounds isolated from Tianma; Figures S11–S16: UHPLC chromatogram for purity detection of compound 1; Figures S17–S48: NMR spectra of compound **1**–**6**; Table S1: Cell viability of glutamate-induced HT-22 cells treated by compounds **5** and **11**; Table S2: Cell viability of glutamate-induced HT-22 cells treated by compounds **1** and **2**.
