# Supplementary material for: Six New Phenolic Compounds in Ethyl Acetate Extract of Tall Gastrodia Tuber (Tianma) with Four Compounds Screened Preliminarily for Cytoprotective Effects Against Excitotoxicity
Source: Pharmaceuticals (Basel). 2026 Jul 10;19(7):1068. doi: 10.3390/ph19071068 (PMC13414891; doi:10.3390/ph19071068)
Supplement: Supplementary file 1 [file pharmaceuticals-19-01068-s001.zip › pharmaceuticals-4335519-supplementary.pdf]

Supplementary material

Six new phenolic compounds in ethyl acetate extract of Tall Gastrodia Tuber (Tianma) with four compounds screened preliminary for cytoprotective effects against excitotoxicity

Contents of supplemental material

Figure S1. UV spectra of six new compounds isolated from Tianma.

Figure S2. FT-IR spectrum of compound **1**, gastrotribenzyloside A.

Figure S3. FT-IR spectrum of compound **2**, gastrotribenzyloside B.

Figure S4. FT-IR spectrum of compound **3**, gastrotribenzyloside C.

Figure S5. FT-IR spectrum of compound **4**, gastrotetrabenzyloside D.

Figure S6. FT-IR spectrum of compound **5**, gastronucleoside B.

Figure S7. FT-IR spectrum of compound **6**, gastronucleoside C.

Figure S8. MS^2 fragments and neutral losses of typical compounds reported in Tianma.

Figure S9. HRMS spectra of six new compounds isolated from Tianma.

Figure S10. MS^2 spectra of six new compounds isolated from Tianma.

Figure S11. UHPLC chromatogram for purity detection of compound **1**.

Figure S12. UHPLC chromatogram for purity detection of compound **2**.

Figure S13. UHPLC chromatogram for purity detection of compound **3**.

Figure S14. UHPLC chromatogram for purity detection of compound **4**.

Figure S15. UHPLC chromatogram for purity detection of compound **5**.

Figure S16. UHPLC chromatogram for purity detection of compound **6**.

Figure S17. ^{1}H-NMR spectrum of compound **1**, gastrotribenzyloside A.

Figure S18. ^{13}C-NMR spectrum of compound **1**, gastrotribenzyloside A.

Figure S19. DEPT 90 and 135 spectra of compound **1**, gastrotribenzyloside A.

Figure S20. ^{1}H-^{1}H COSY spectrum of compound **1**, gastrotribenzyloside A.

Figure S21. HSQC spectrum of compound **1**, gastrotribenzyloside A.

Figure S22. HMBC spectrum of compound **1**, gastrotribenzyloside A.

Figure S23. ^{1}H-NMR spectrum of compound **2**, gastrotribenzyloside B.

Figure S24. ^{13}C-NMR spectrum of compound **2**, gastrotribenzyloside B.

Figure S25. ^{1}H-^{1}H COSY spectrum of compound **2**, gastrotribenzyloside B.

Figure S26. HSQC spectrum of compound **2**, gastrotribenzyloside B.

Figure S27. HMBC spectrum of compound **2**, gastrotribenzyloside B.

Figure S28. ^{1}H-NMR spectrum of compound **3**, gastrotribenzyloside C.

Figure S29. ^{13}C-NMR spectrum of compound **3**, gastrotribenzyloside C.

Figure S30. ^{1}H-1H COSY spectrum of compound **3**, gastrotribenzyloside C.

Figure S31. HSQC spectrum of compound **3**, gastrotribenzyloside C.

Figure S32. HMBC spectrum of compound **3**, gastrotribenzyloside C.

Figure S33. ^{1}H-NMR spectrum of compound **4**, gastrotetrabenzyloside D.

Figure S34. ^{13}C-NMR spectrum of compound **4**, gastrotetrabenzyloside D.

Figure S35. HSQC spectrum of compound **4**, gastrotetrabenzyloside D.

Figure S36. HMBC spectrum of compound **4**, gastrotetrabenzyloside D.

Figure S37. ^{1}H-NMR spectrum of compound **5**, gastronucleoside B.

Figure S38. ^{13}C-NMR spectrum of compound **5**, gastronucleoside B.

Figure S39. DEPT 90 and 135 spectra of compound **5**, gastronucleoside B.

Figure S40. ^{1}H-^{1}H COSY spectrum of compound **5**, gastronucleoside B.

Figure S41. HSQC spectrum of compound **5**, gastronucleoside B.

Figure S42. HMBC spectrum of compound **5**, gastronucleoside B.

Figure S43. ^{1}H-NMR spectrum of compound **6**, gastronucleoside C.

Figure S44. ^{13}C-NMR spectrum of compound **6**, gastronucleoside C.

Figure S45. DEPT 90 and 135 spectra of compound **6**, gastronucleoside C.

Figure S46. ^{1}H-^{1}H COSY spectrum of compound **6**, gastronucleoside C.

Figure S47. HSQC spectrum of compound **6**, gastronucleoside C.

Figure S48. HMBC spectrum of compound **6**, gastronucleoside C.

Table S1. Cell viability of glutamate-induced HT-22 cells treated by compounds **5** and **11**.

Table S2. Cell viability of glutamate-induced HT-22 cells treated by compounds **1** and **2**.

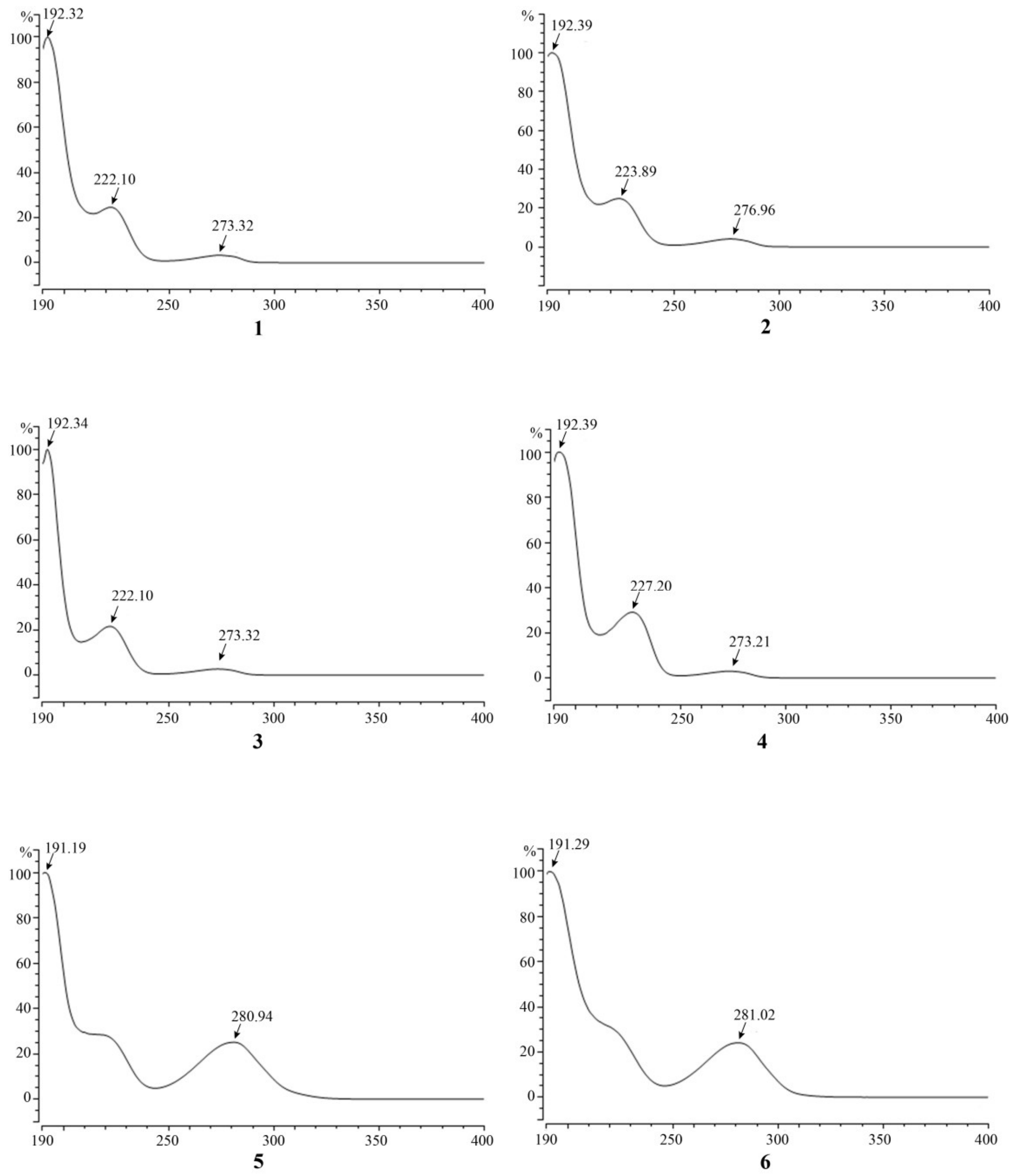

Figure S1. UV spectra of the six new compounds isolated from Tianma. (**1**) gastrotribenzyloside A; (**2**) gastrotribenzyloside B; (**3**) gastrotribenzyloside C; (**4**) gastrotetrabenzyloside D; (**5**) gastronucleoside B; (**6**) gastronucleoside C.

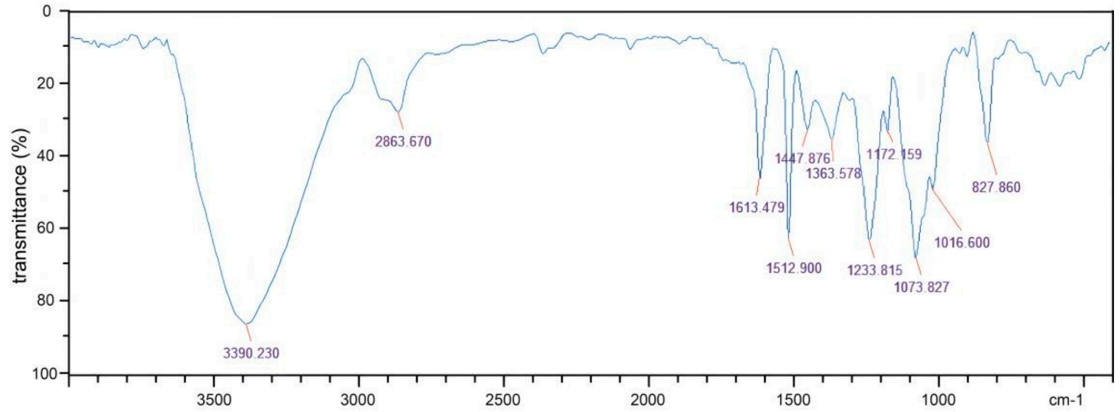

Figure S2. FT-IR spectrum of compound **1**, gastrotribenzyloside A.

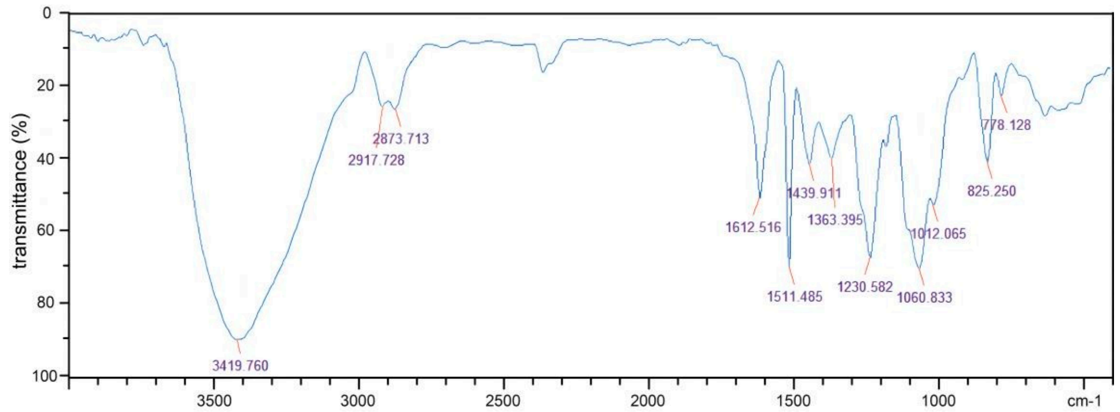

Figure S3. FT-IR spectrum of compound **2**, gastrotribenzyloside B.

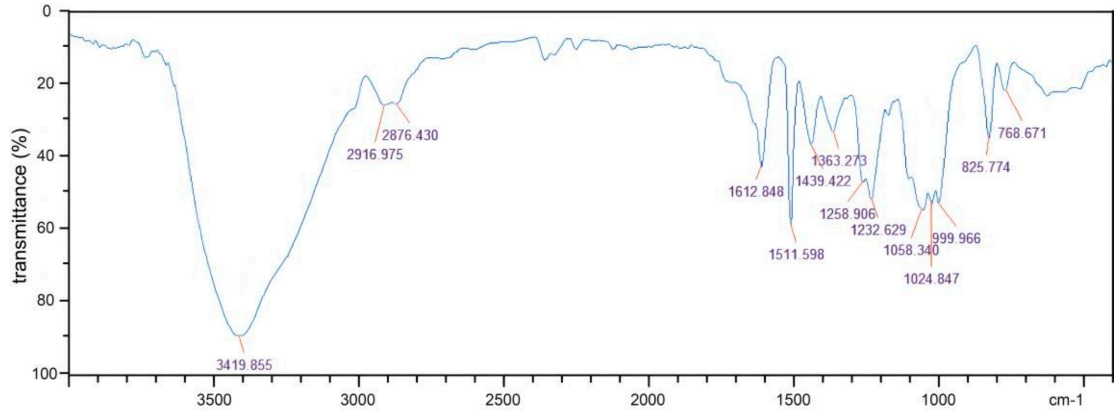

Figure S4. FT-IR spectrum of compound **3**, gastrotribenzyloside C.

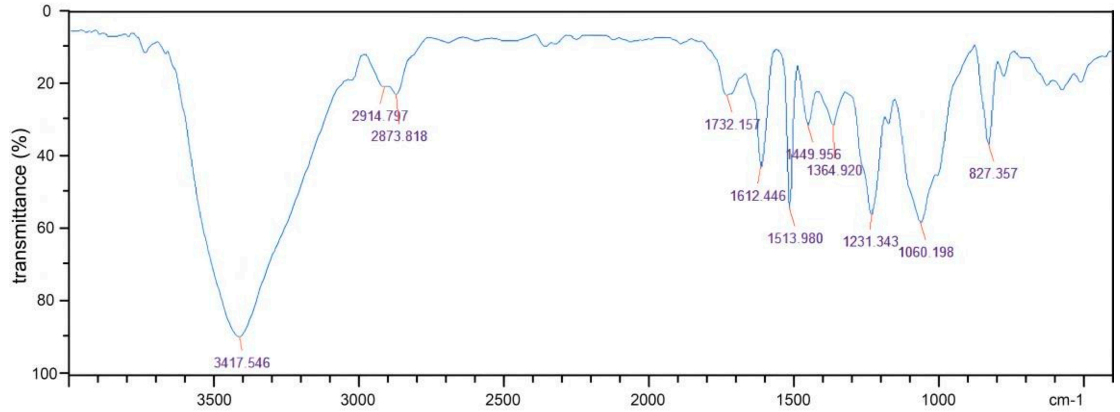

Figure S5. FT-IR spectrum of compound **4**, gastrotetrabenzyloside D.

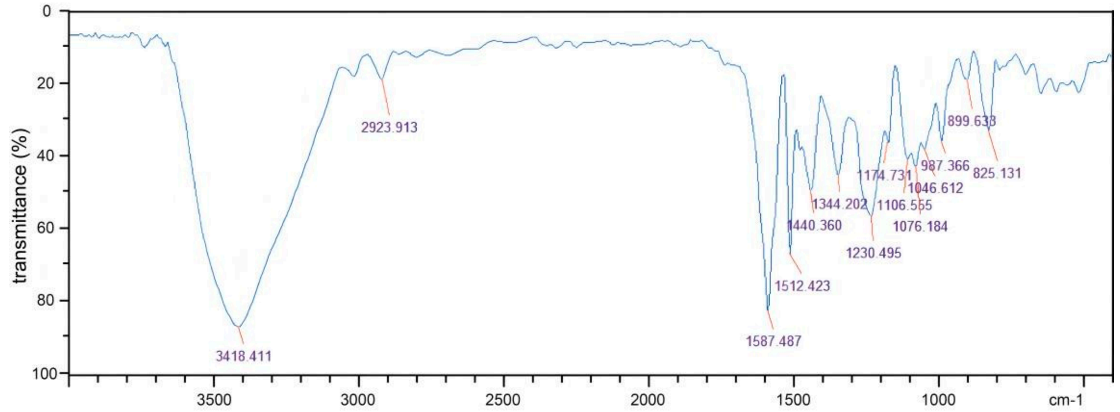

Figure S6. FT-IR spectrum of compound **5**, gastronucleoside B.

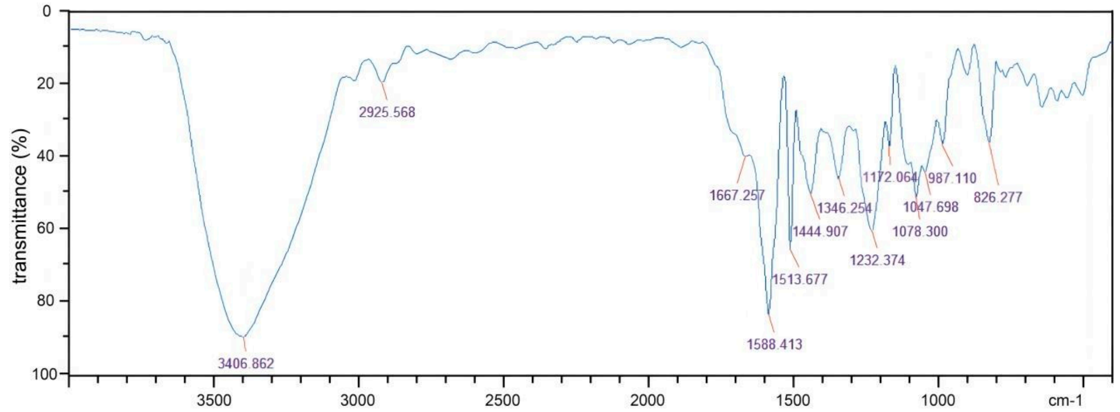

Figure S7. FT-IR spectrum of compound **6**, gastronucleoside C.

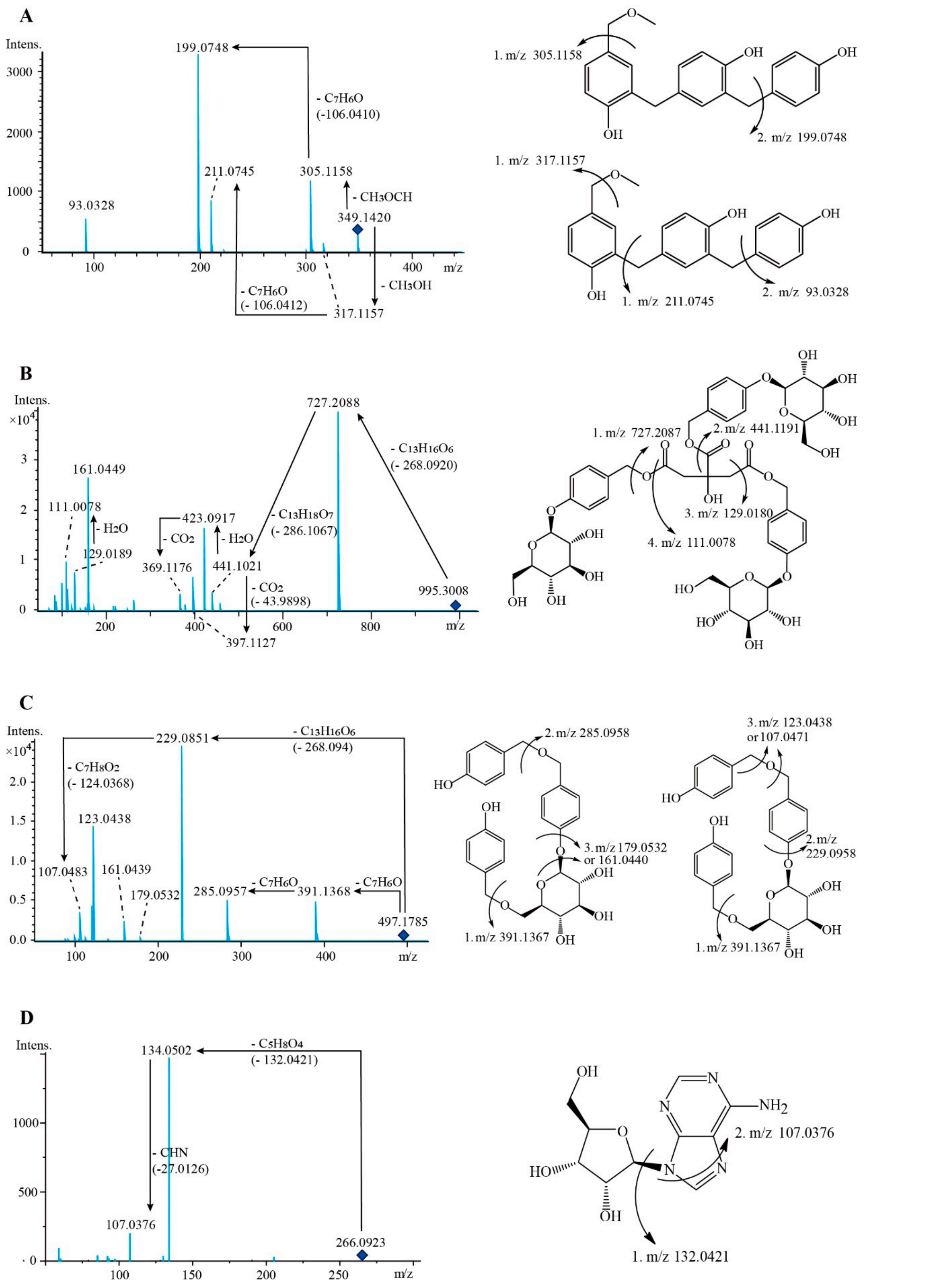

Figure S8. MS^2 fragments and neutral losses of typical compounds reported in Tianma. (A) 2-[3-(4-hydroxybenzyl)-4-hydroxybenzyl]-4-methoxymethyl benzyl, [89] (B) parishin B, (C) 6',7-di-O-(4- hydroxybenzyl) gastrodin and (D) adenosine.

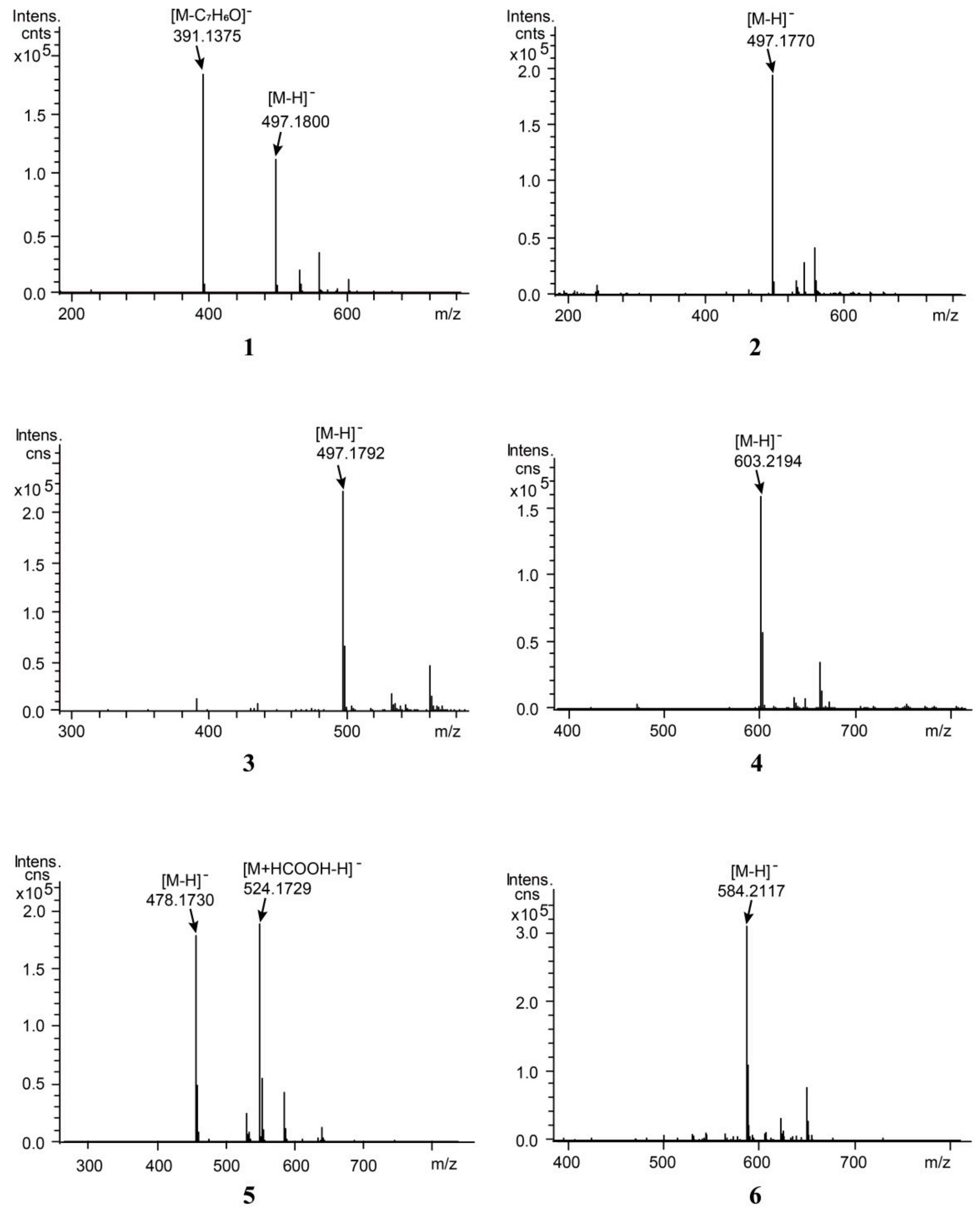

Figure S9. HRMS spectra of six new compounds isolated from Tianma. (**1**) gastrotribenzyloside A; (**2**) gastrotribenzyloside B; (**3**) gastrotribenzyloside C; (**4**) gastrotetrabenzyloside D; (**5**) gastronucleoside B; (**6**) gastronucleoside C.

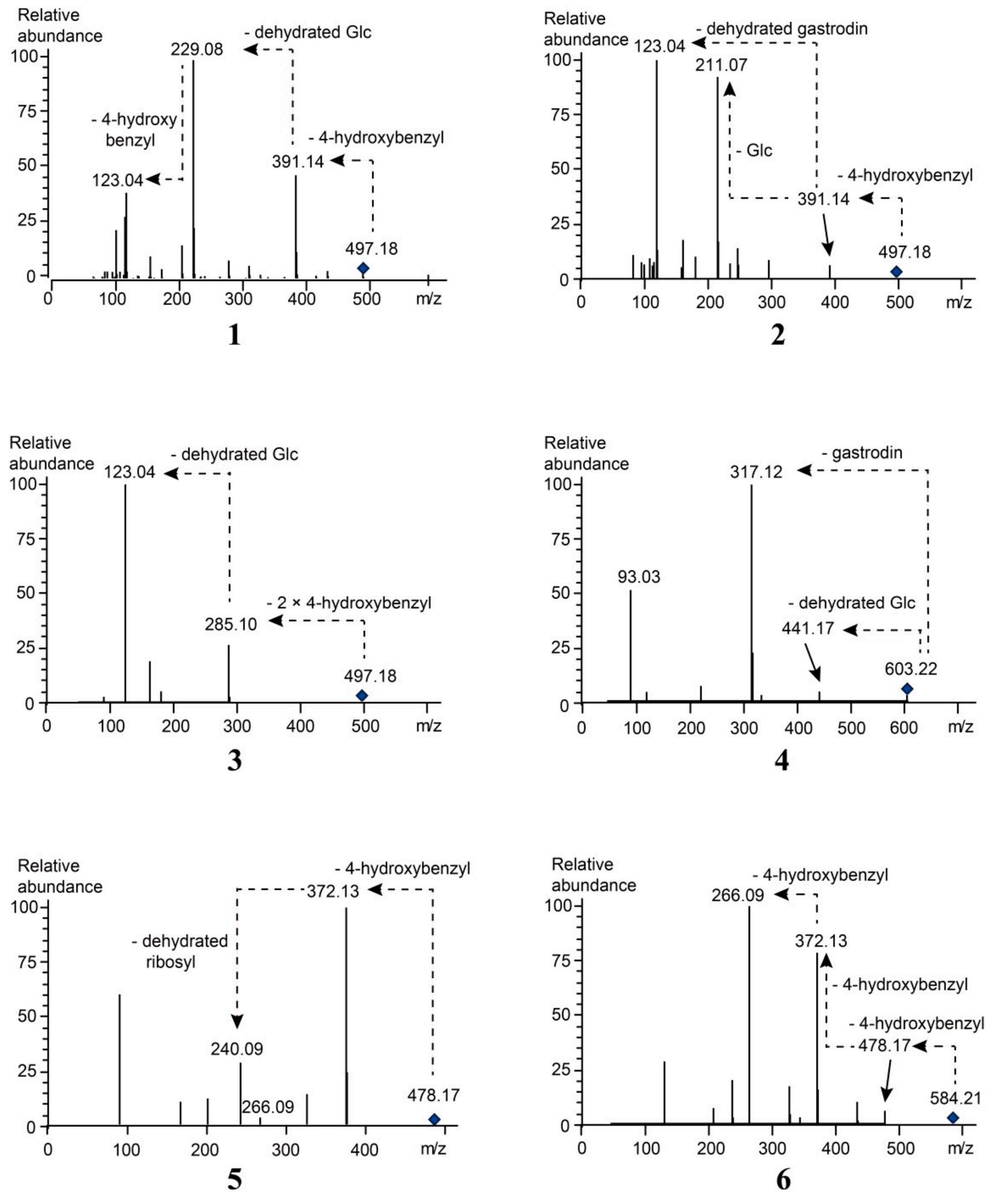

Figure S10. MS^2 spectra and fragmentation pathways of the six new compounds isolated from Tianma. (**1**) gastrotribenzyloside A; (**2**) gastrotribenzyloside B; (**3**) gastrotribenzyloside C; (**4**) gastrotetrabenzyloside D; (**5**) gastronucleoside B; (**6**) gastronucleoside C.

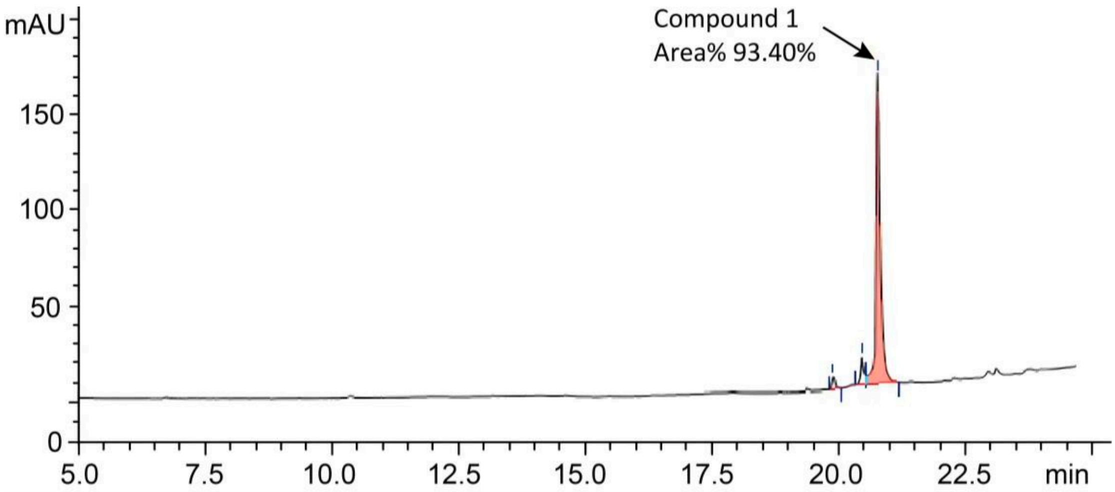

Figure S11. UHPLC chromatogram for purity detection of compound **1** (280 nm).

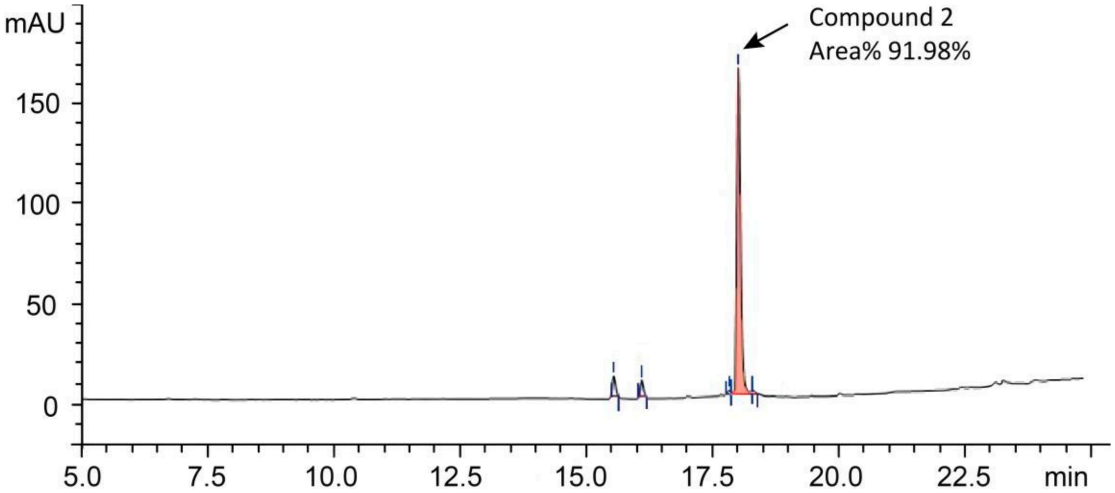

Figure S12. UHPLC chromatogram for purity detection of compound **2** (280 nm).

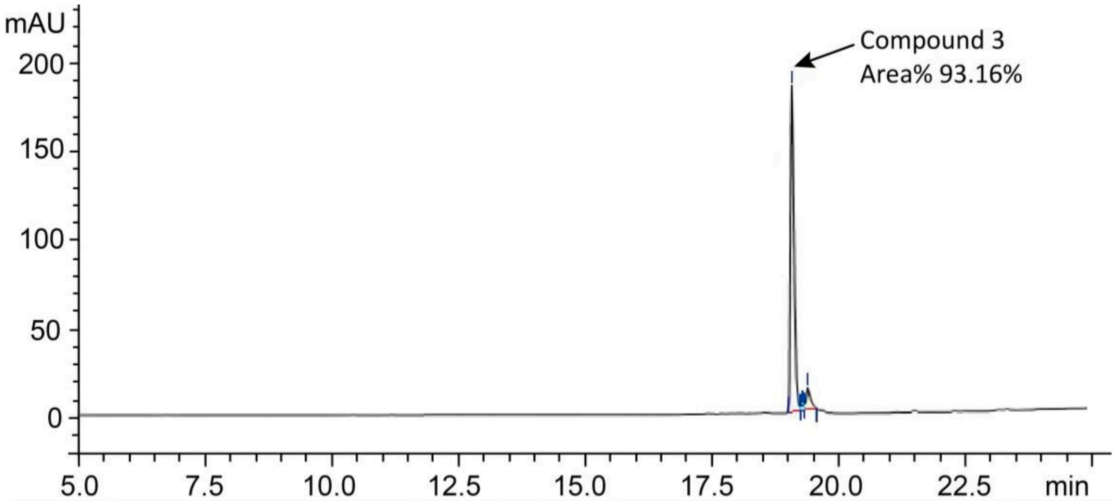

Figure S13. UHPLC chromatogram for purity detection of compound **3** (280 nm).

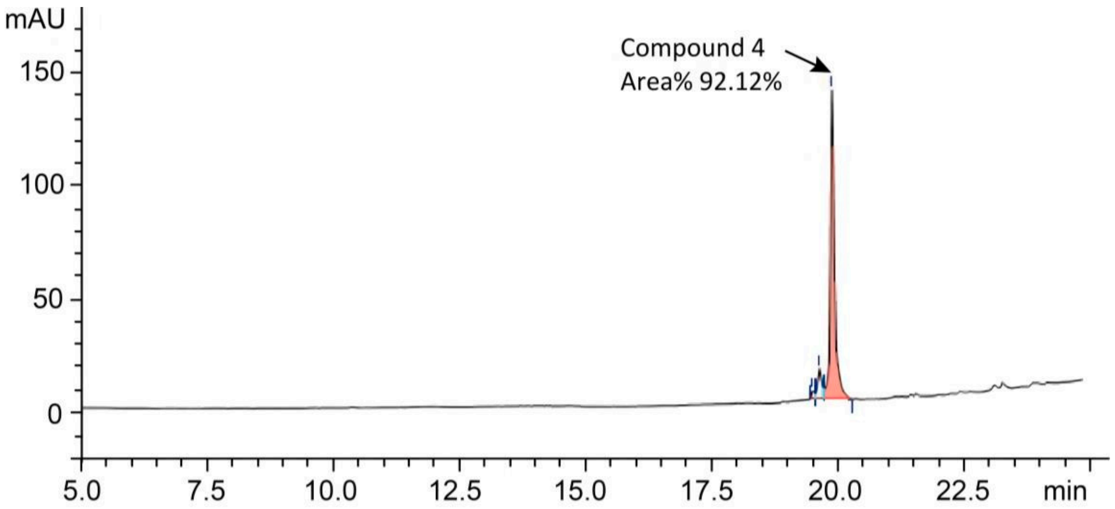

Figure S14. UHPLC chromatogram for purity detection of compound **4** (280 nm).

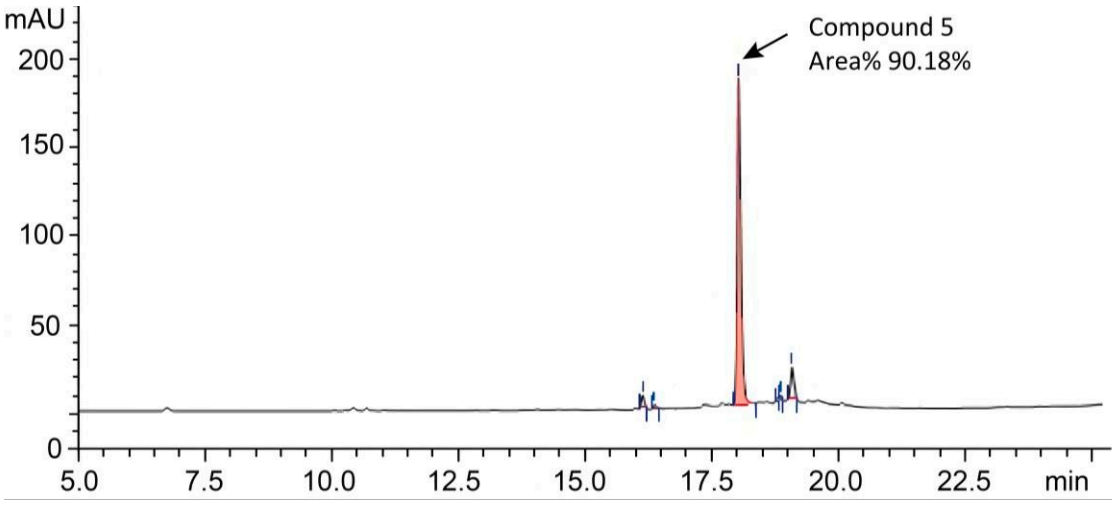

Figure S15. UHPLC chromatogram for purity detection of compound **5** (280 nm).

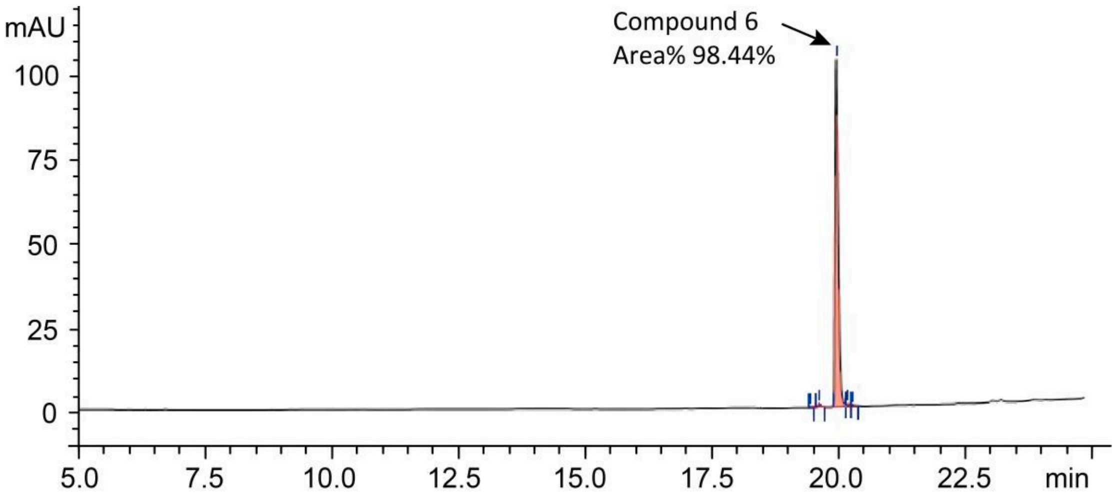

Figure S16. UHPLC chromatogram for purity detection of compound **6** (280 nm).

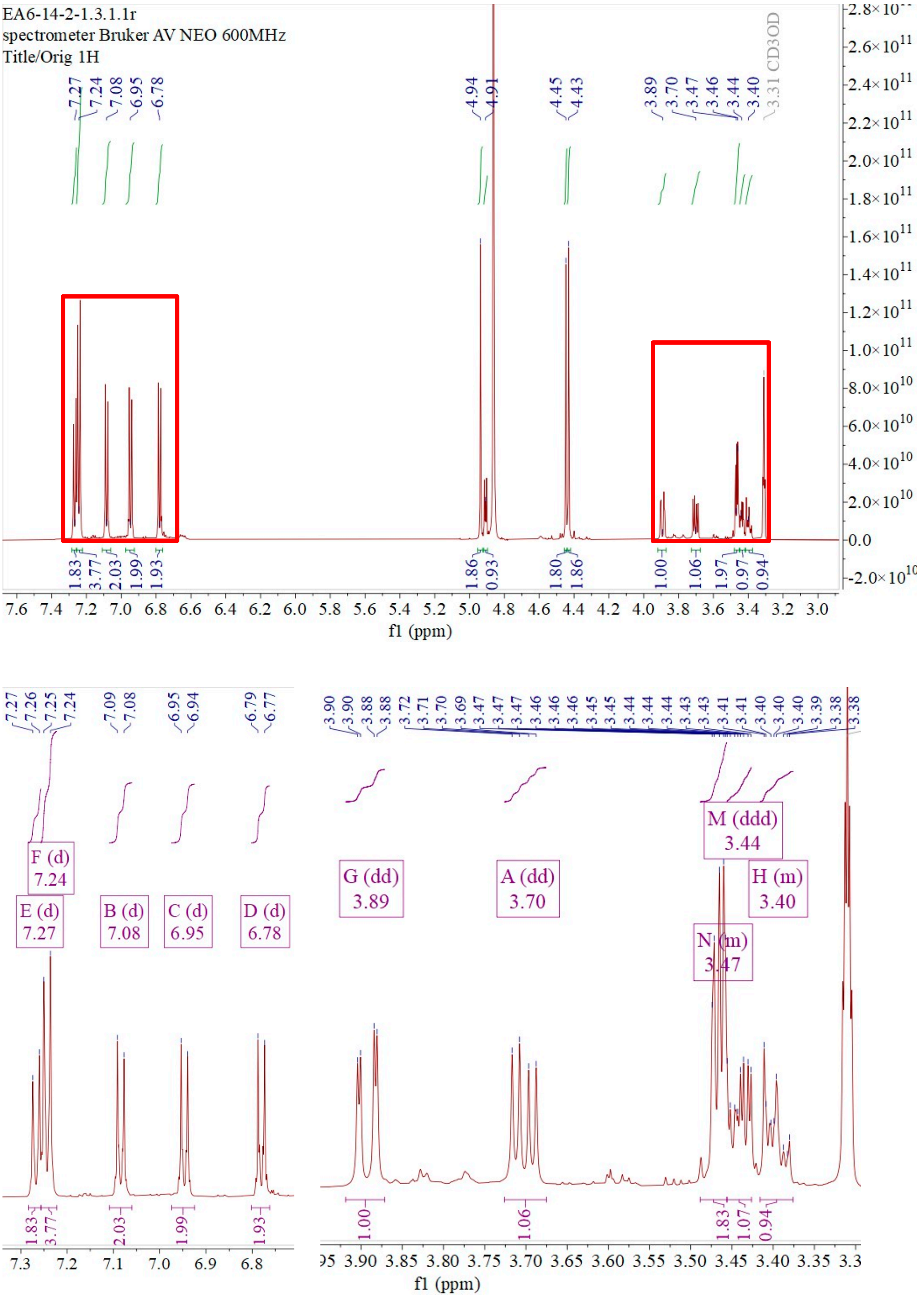

Figure S17. ^{1}H-NMR spectrum of compound **1**, gastrotribenzyloside A (CD_3OD, 600 MHz)

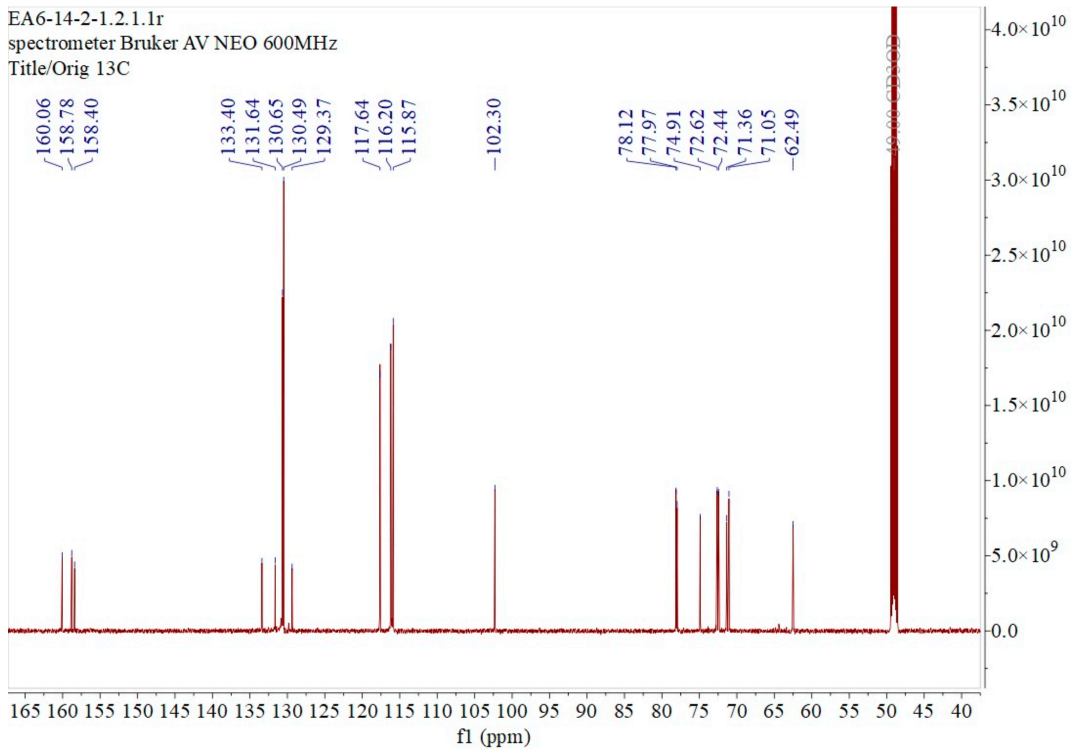

Figure S18. ^{13}C-NMR spectrum of compound **1**, gastrotribenzyloside A (CD$_3$OD, 150 MHz)

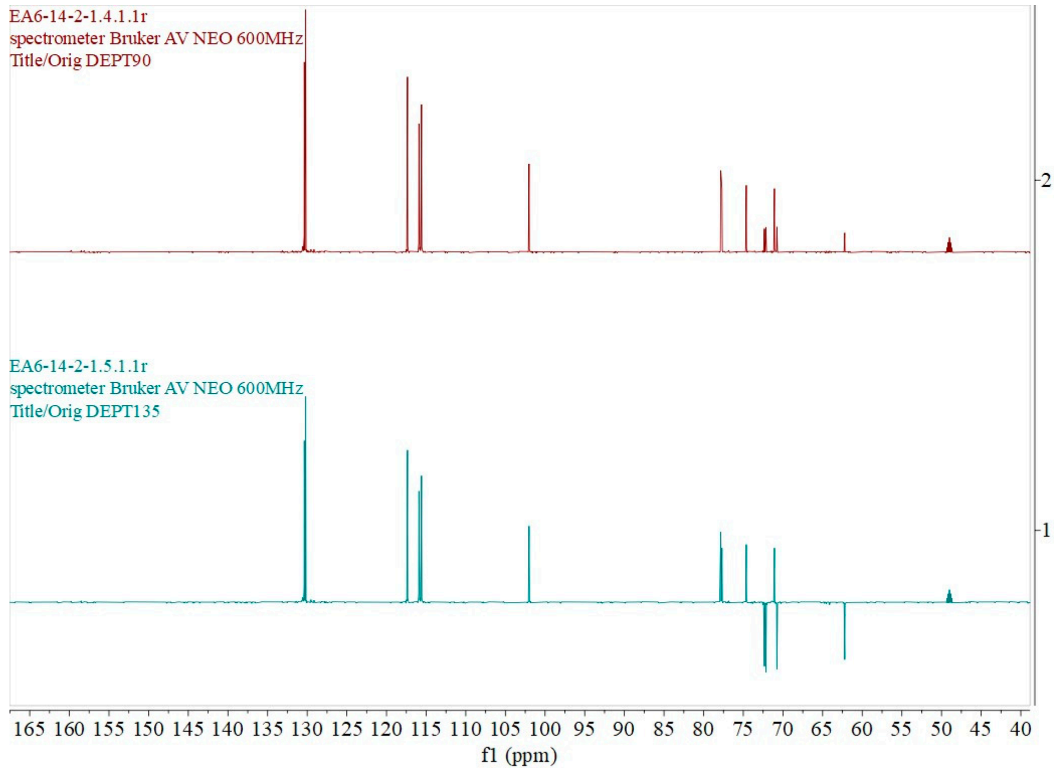

Figure S19. DEPT 90 and 135 spectra of compound **1**, gastrotribenzyloside A (CD$_3$OD, 150 MHz)

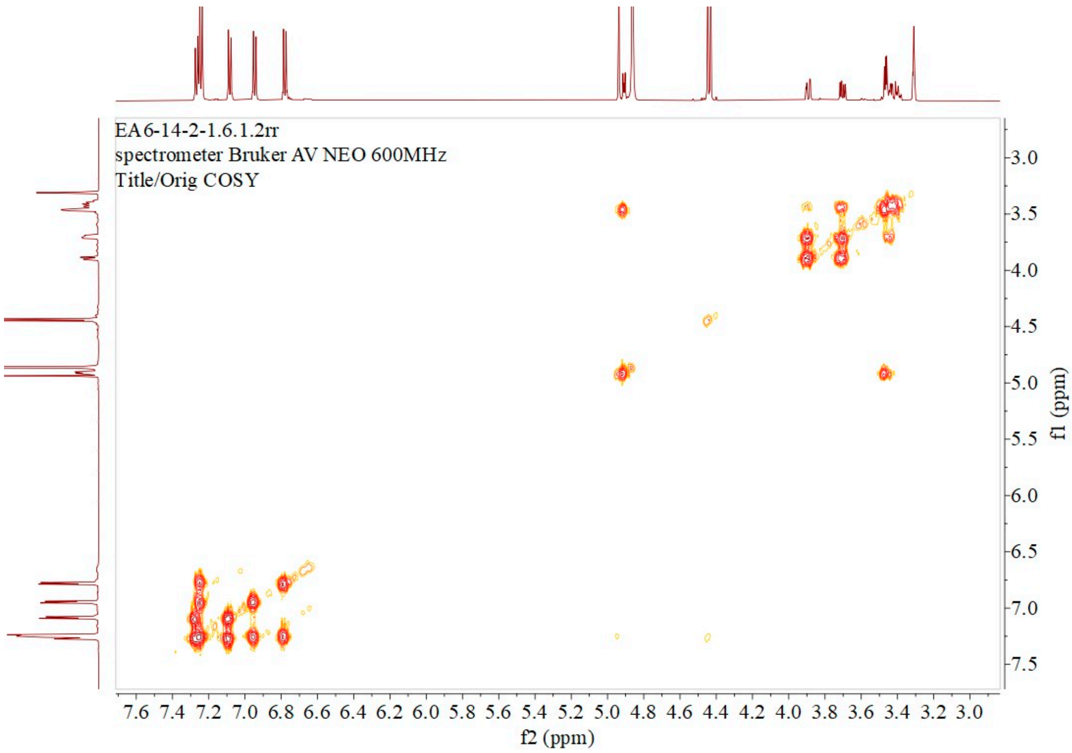

Figure S20. ^{1}H-^{1}H COSY spectrum of compound **1**, gastrotribenzyloside A (CD_3OD)

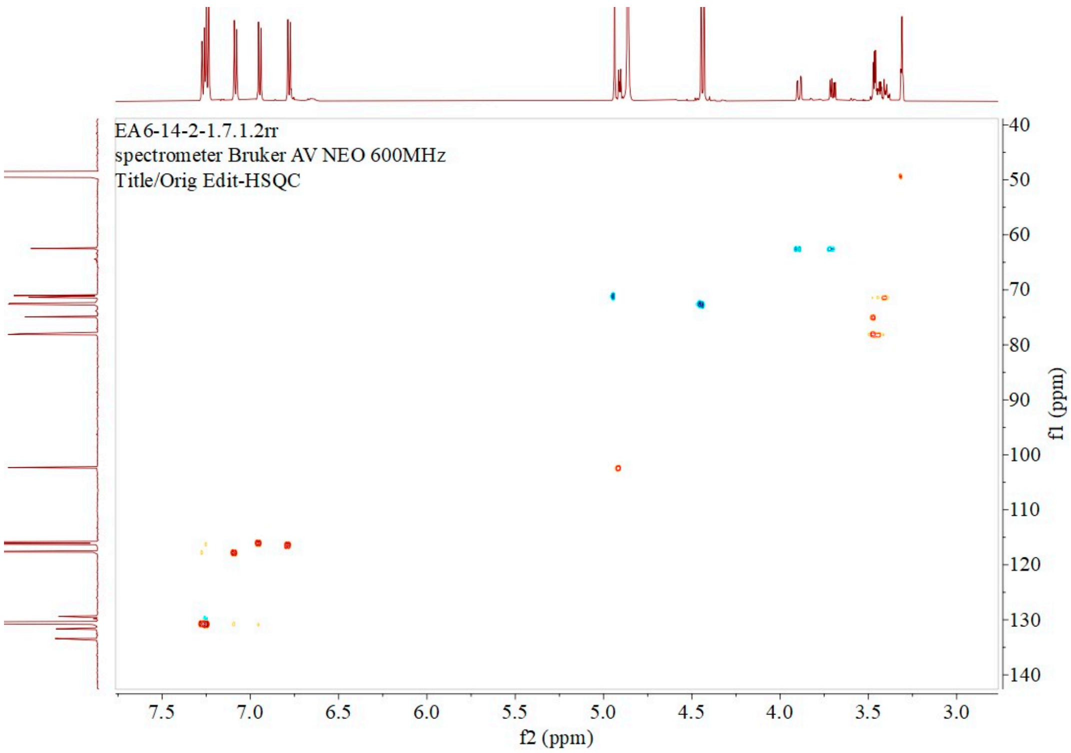

Figure S21. HSQC spectrum of compound **1**, gastrotribenzyloside A (CD_3OD)

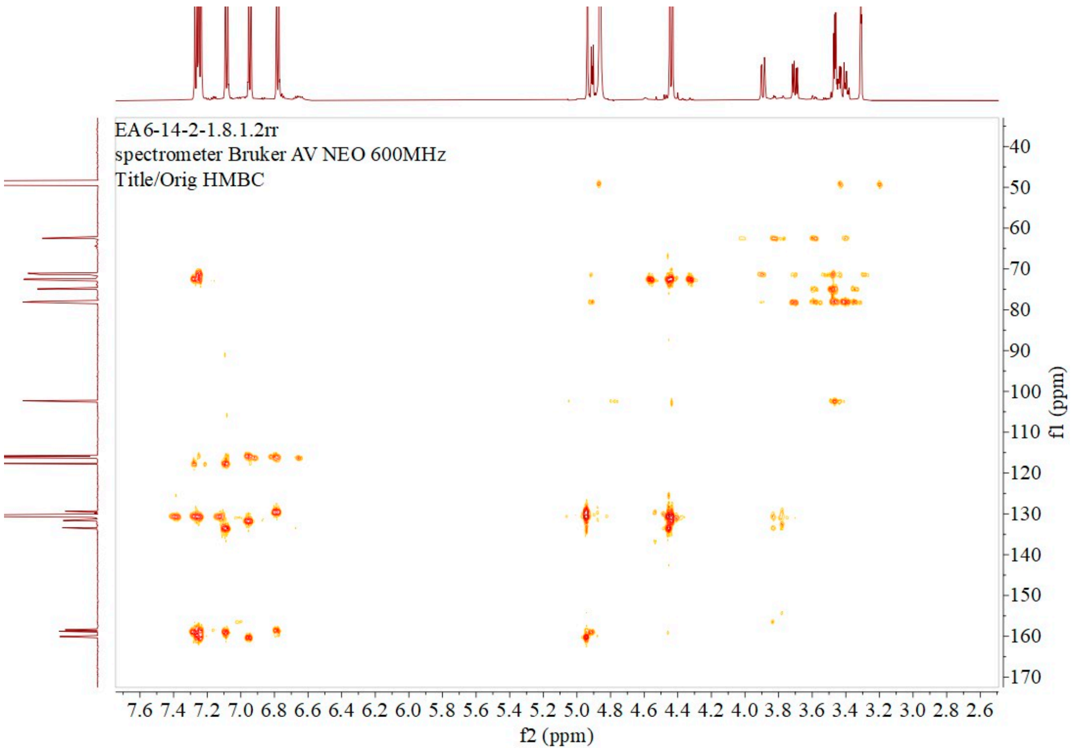

Figure S22. HMBC spectrum of compound **1**, gastrotribenzyloside A (CD_3OD)

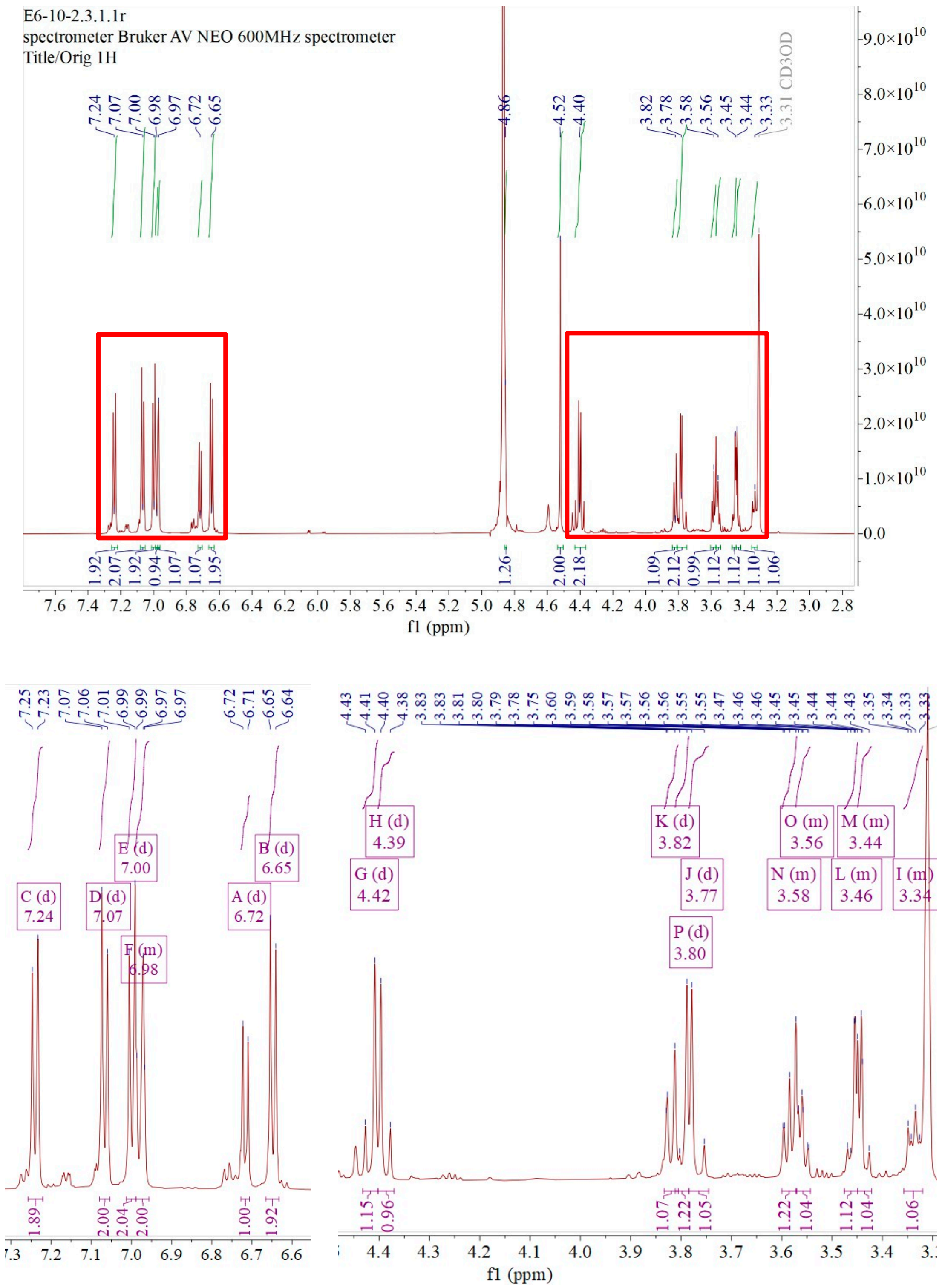

Figure S23. ^{1}H-NMR spectrum of compound **2**, gastrotribenzyloside B (CD_3OD, 600 MHz)

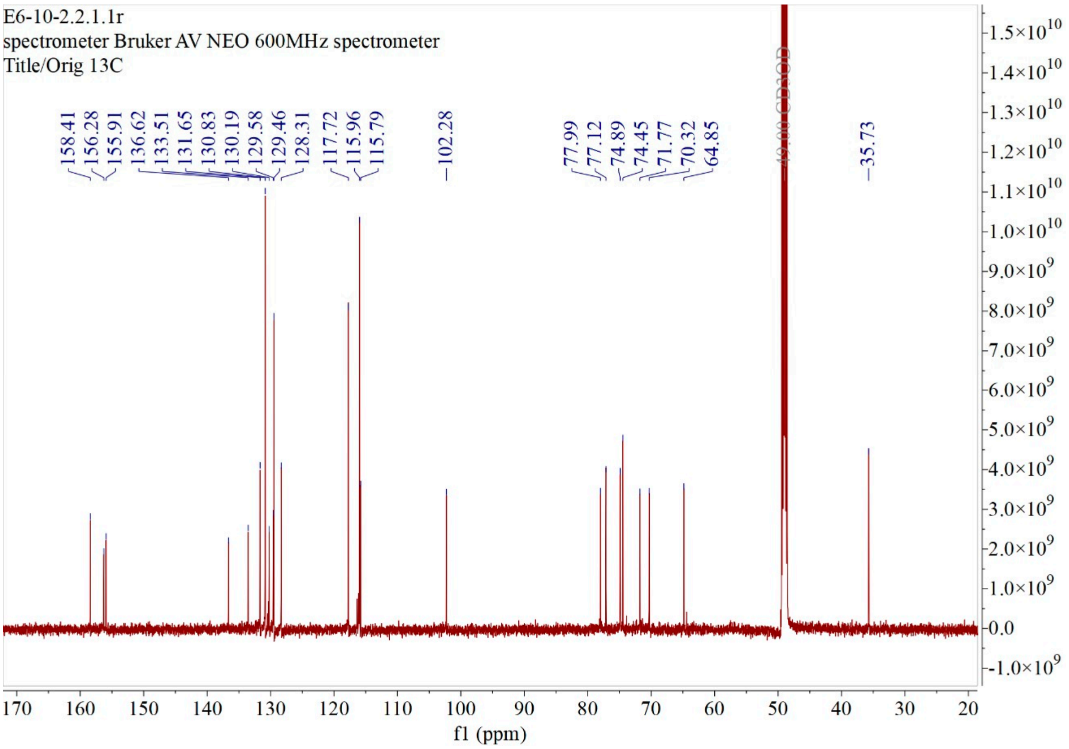

Figure S24. ^{13}C-NMR spectrum of compound **2**, gastrotribenzyloside B (CD_3OD, 150 MHz)

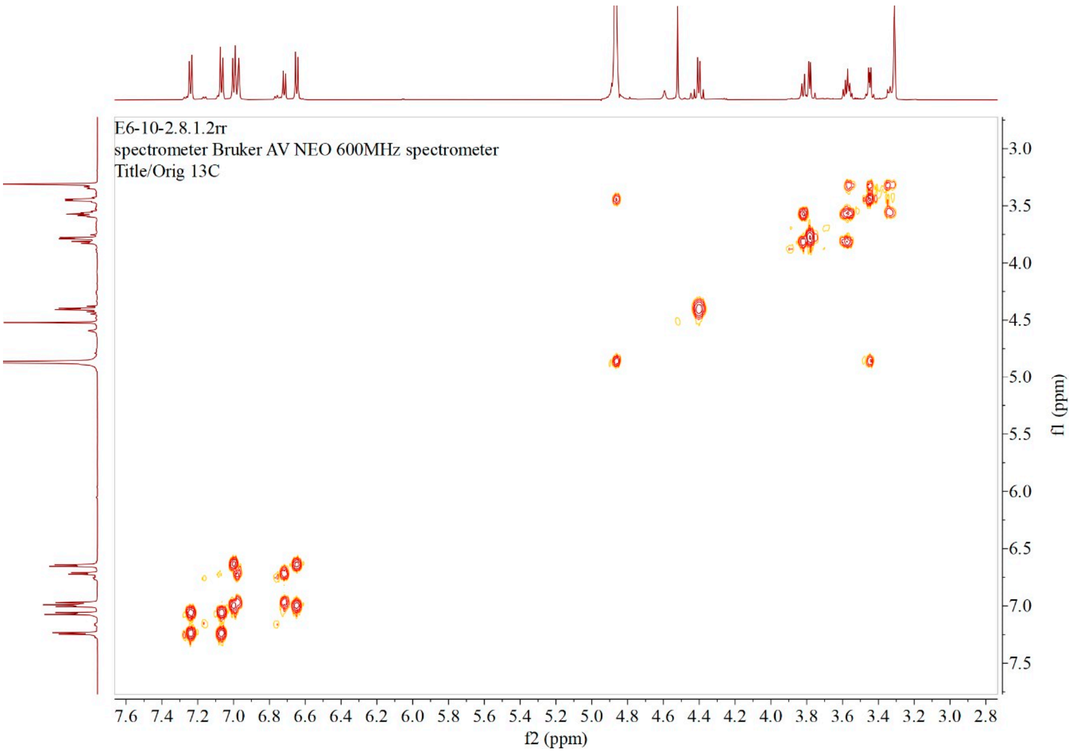

Figure S25. ^{1}H-^{1}H COSY spectrum of compound **2**, gastrotribenzyloside B (CD_3OD)

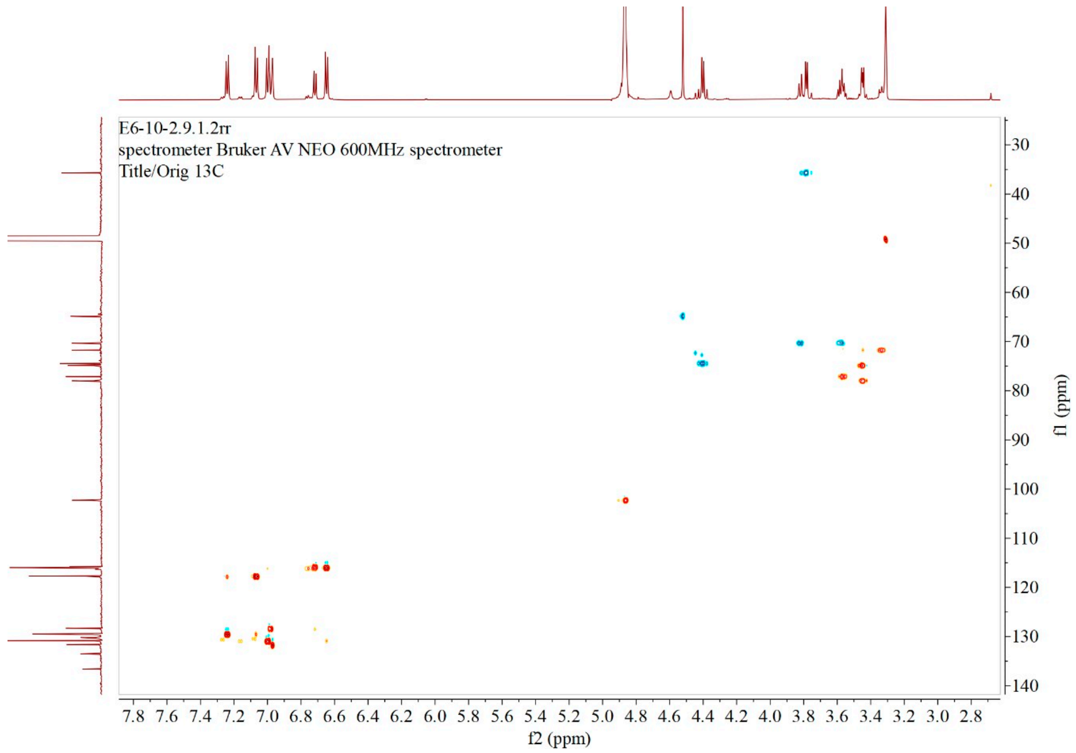

Figure S26. HSQC spectrum of compound **2**, gastrotribenzyloside B (CD_3OD)

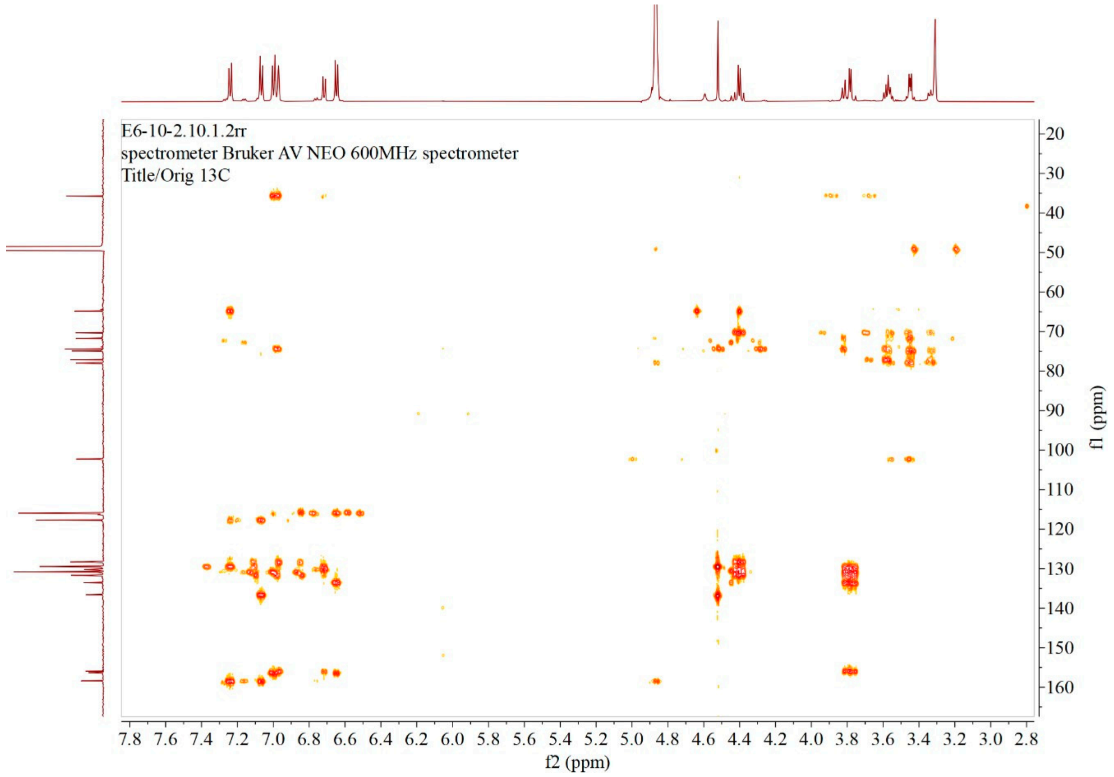

Figure S27. HMBC spectrum of compound **2**, gastrotribenzyloside B (CD_3OD)

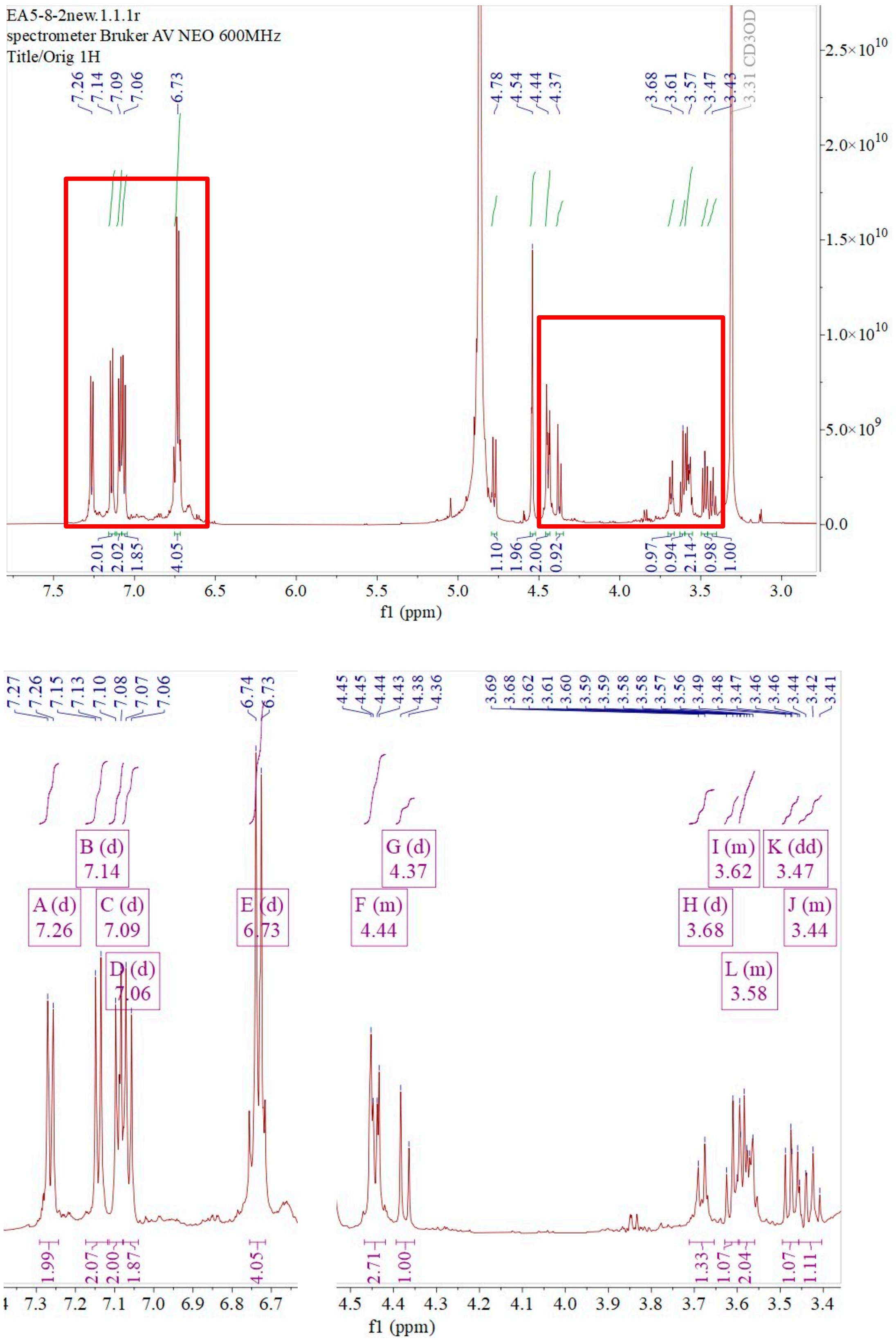

Figure S28. ^{1}H-NMR spectrum of compound **3**, gastrotribenzyloside C (CD_3OD, 600 MHz)

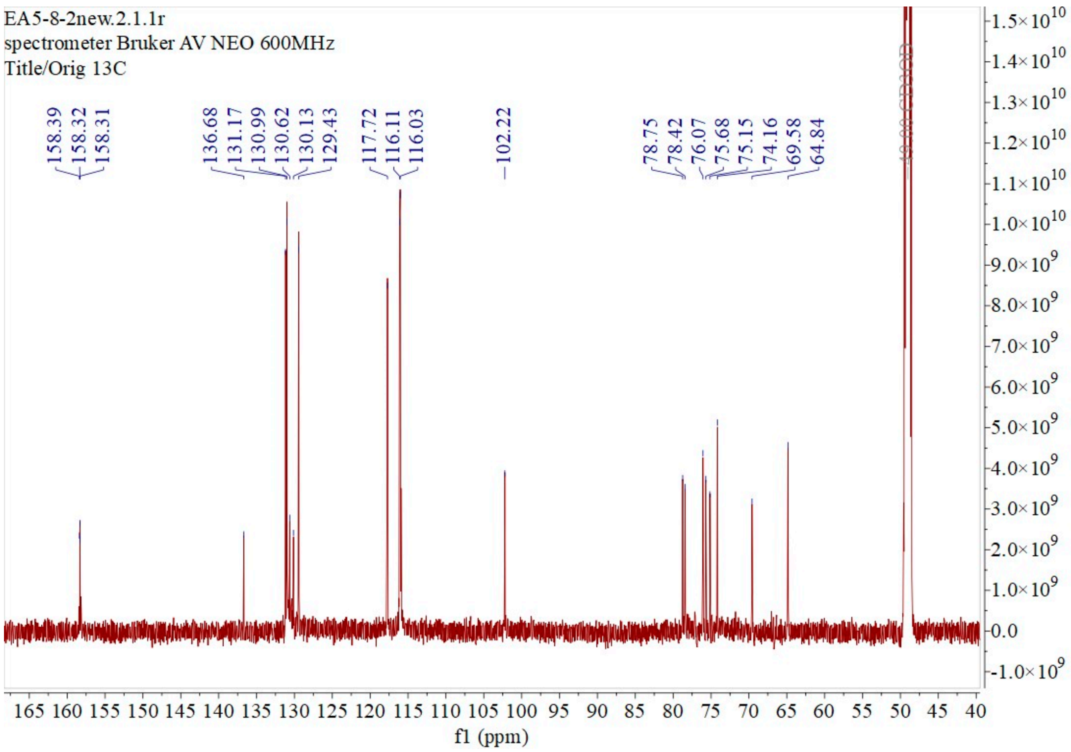

Figure S29. ^{13}C-NMR spectrum of compound **3**, gastrotribenzyloside C (CD_3OD, 150 MHz)

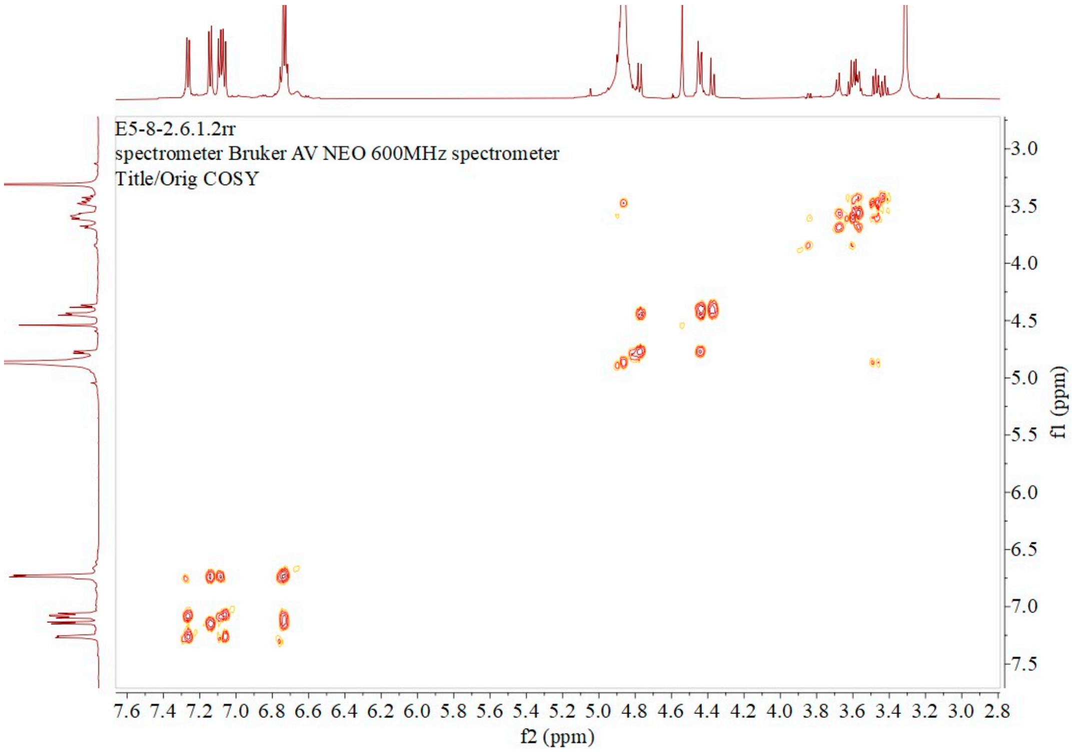

Figure S30. ^{1}H-^{1}H COSY spectrum of compound **3**, gastrotribenzyloside C (CD_3OD)

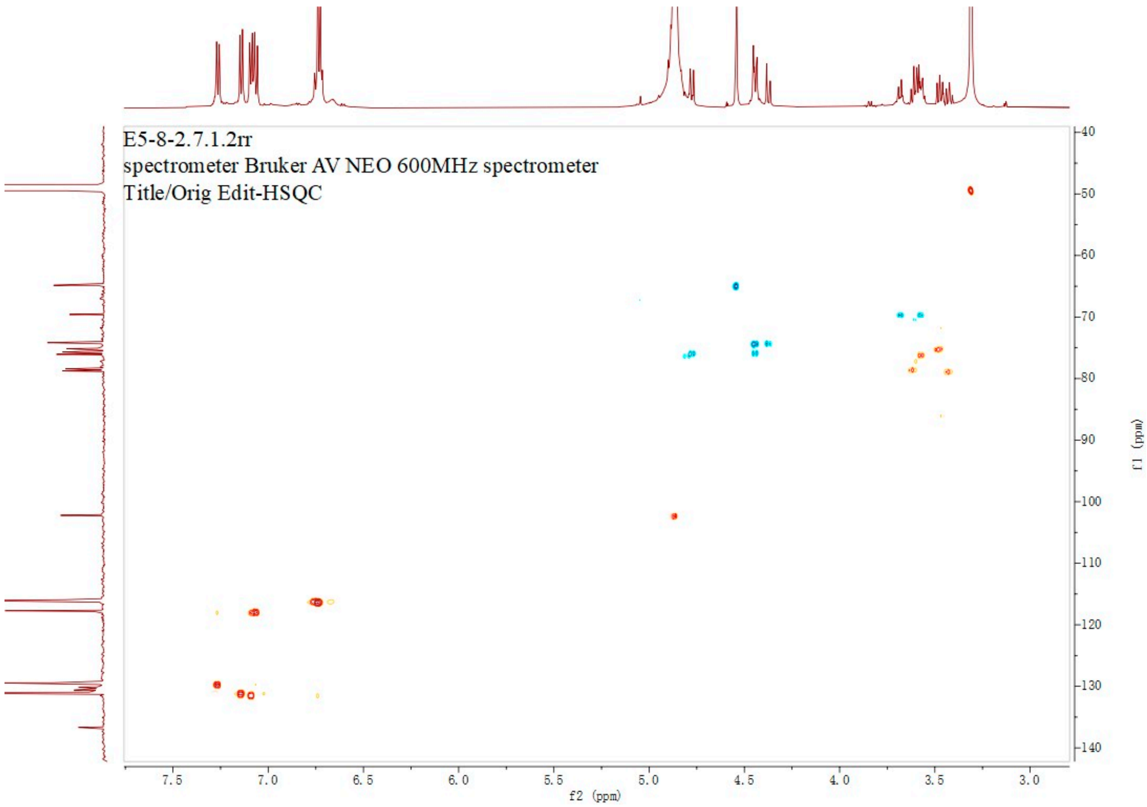

Figure S31. HSQC spectrum of compound **3**, gastrotribenzyloside C (CD_3OD)

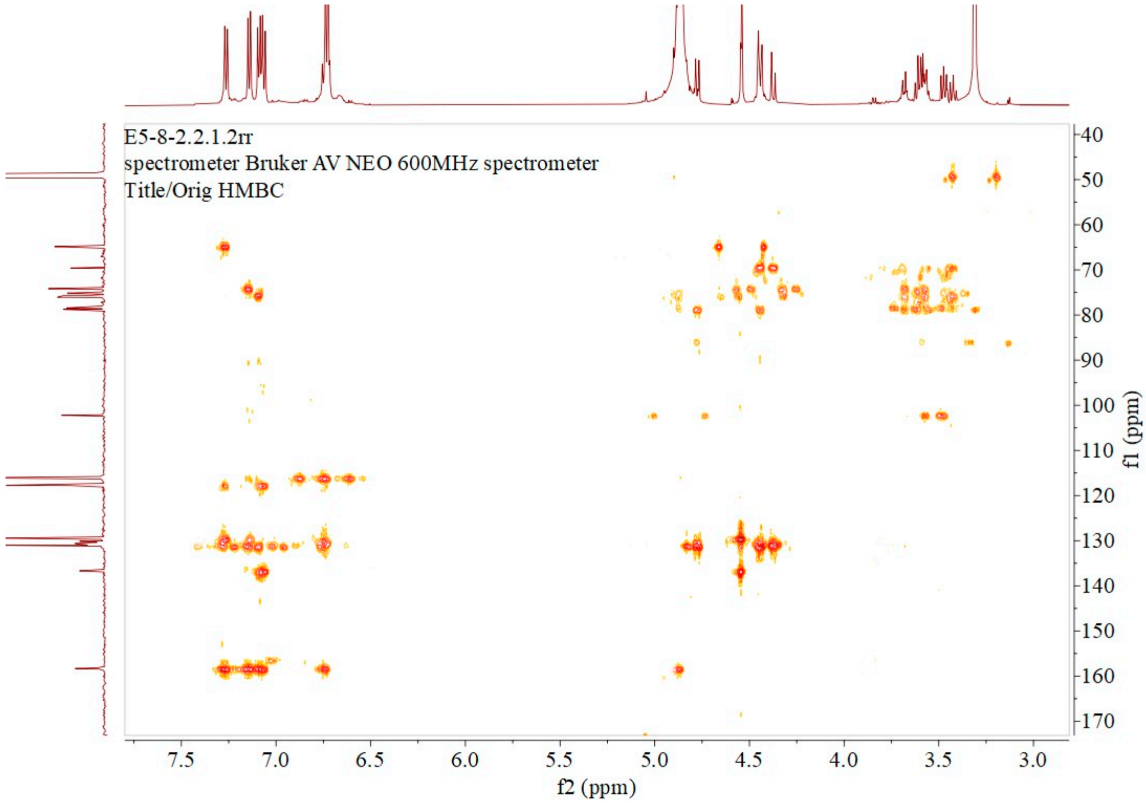

Figure S32. HMBC spectrum of compound **3**, gastrotribenzyloside C (CD_3OD)

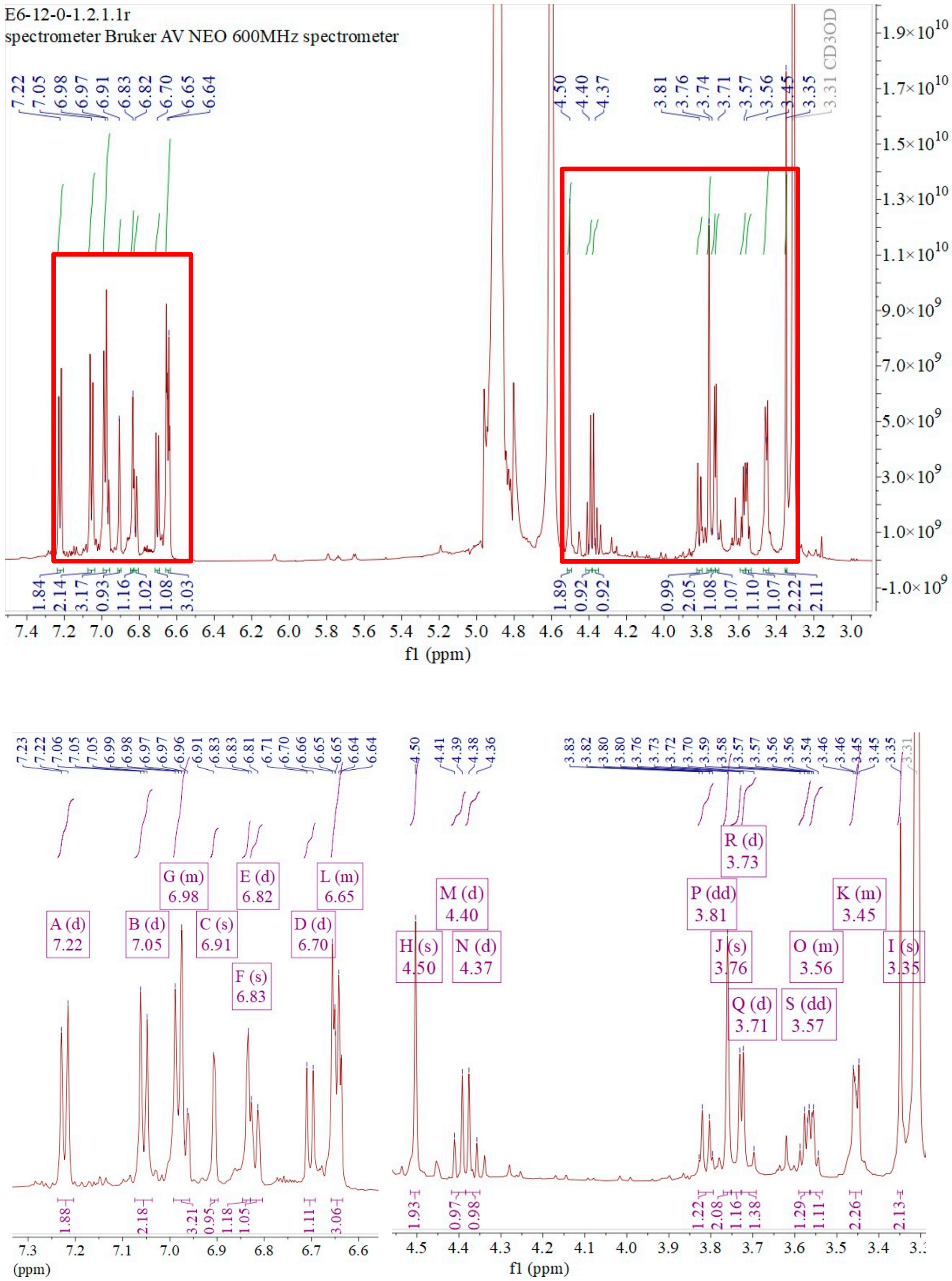

Figure S33. ^{1}H-NMR spectrum of compound **4**, gastrotetrabenzyloside D (CD_3OD, 600 MHz)

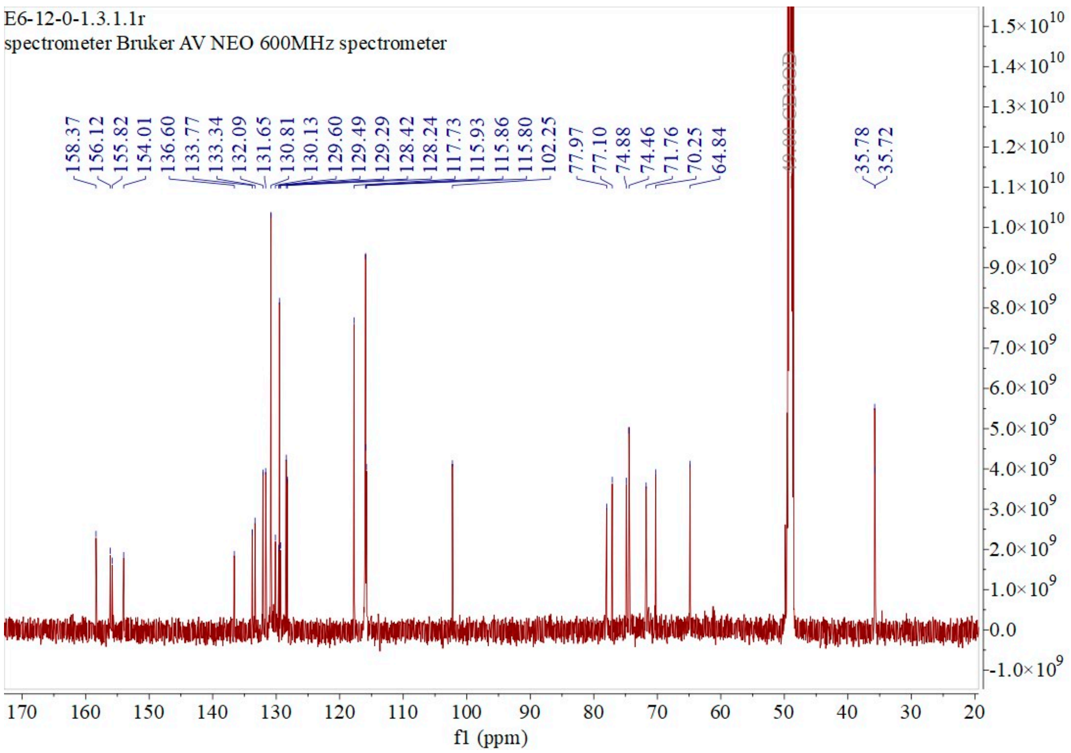

Figure S34. ^{13}C-NMR spectrum of compound **4**, gastrotetrabenzyloside D (CD_3OD, 150 MHz)

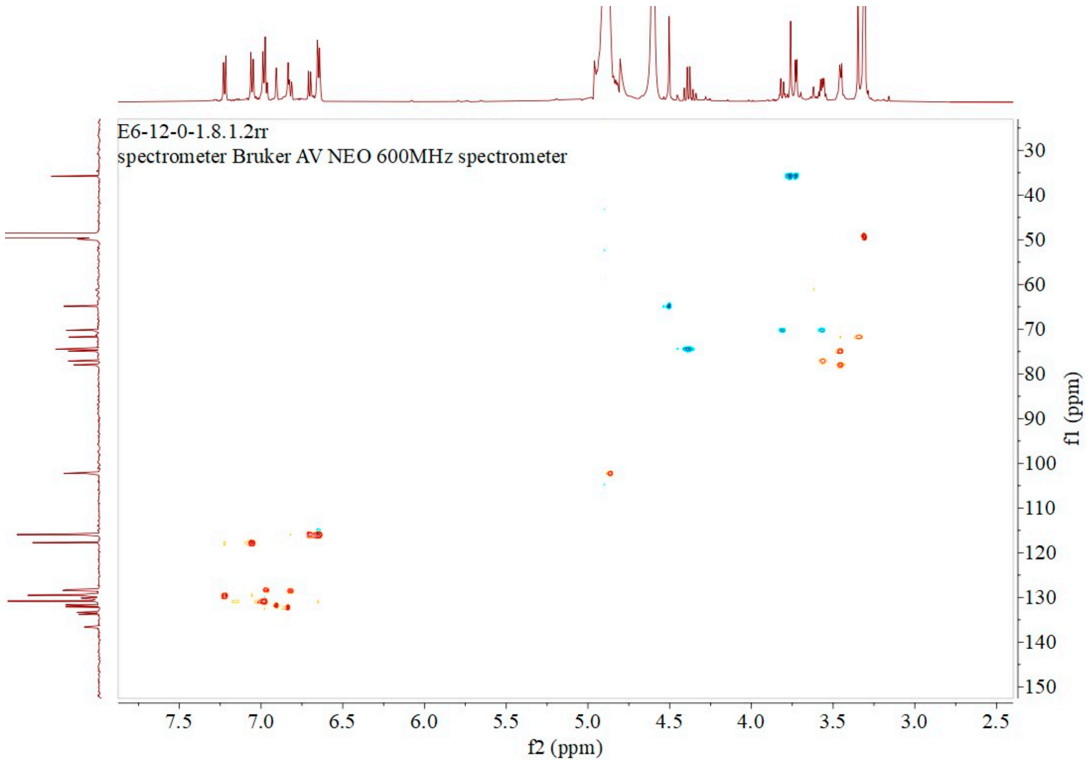

Figure S35. HSQC spectrum of compound **4**, gastrotetrabenzyloside D (CD_3OD)

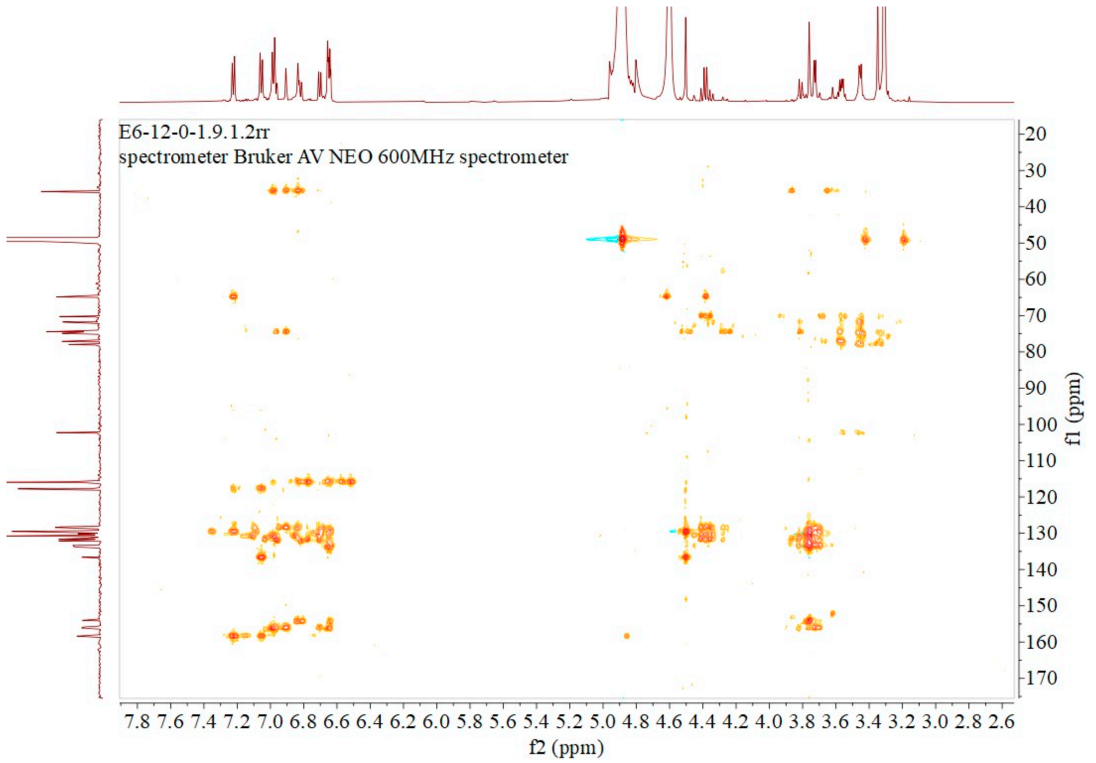

Figure S36. HMBC spectrum of compound **4**, gastrotetrabenzyloside D (CD_3OD)

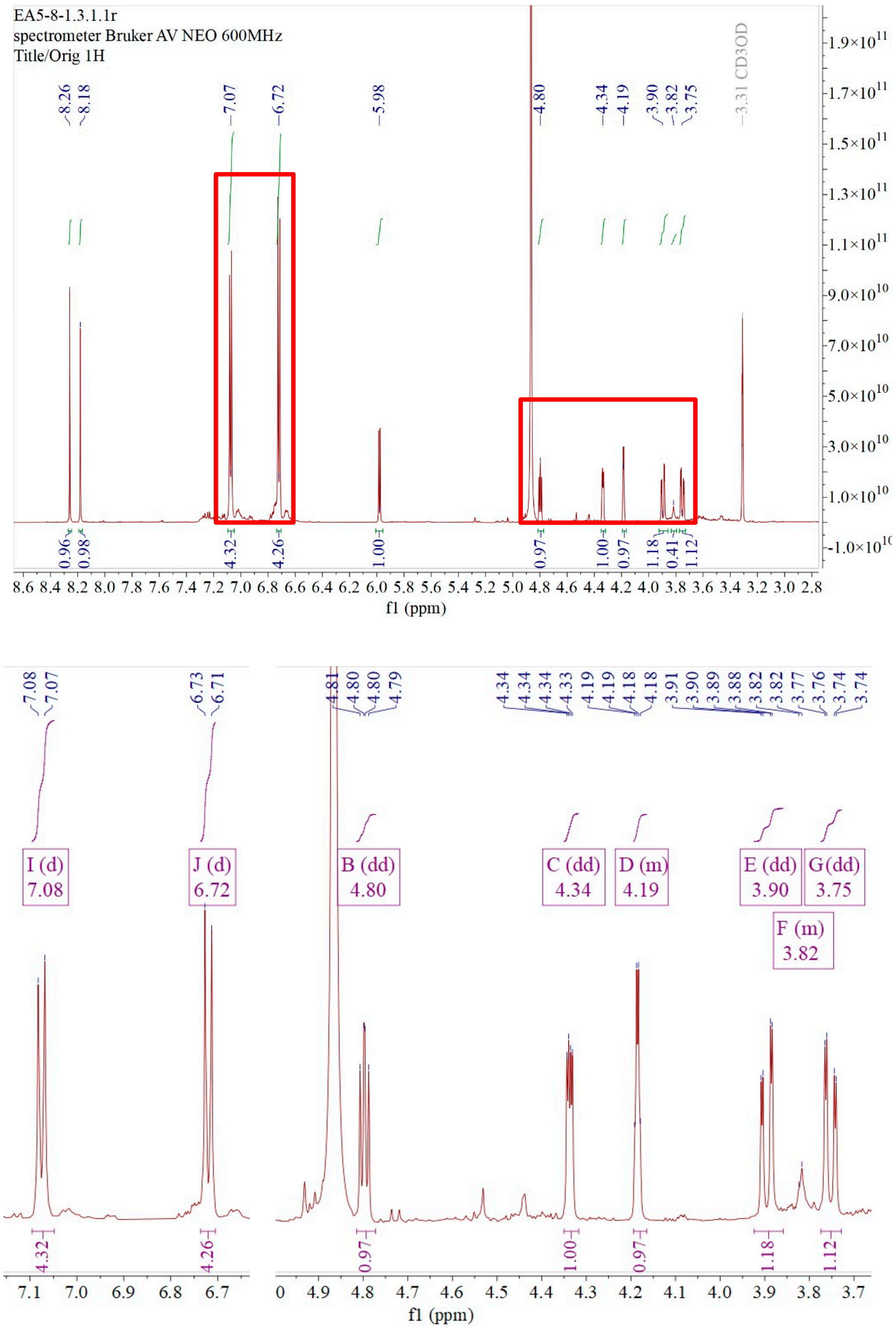

Figure S37. ^{1}H-NMR spectrum of compound **5**, gastronucleoside B (CD_3OD, 600 MHz)

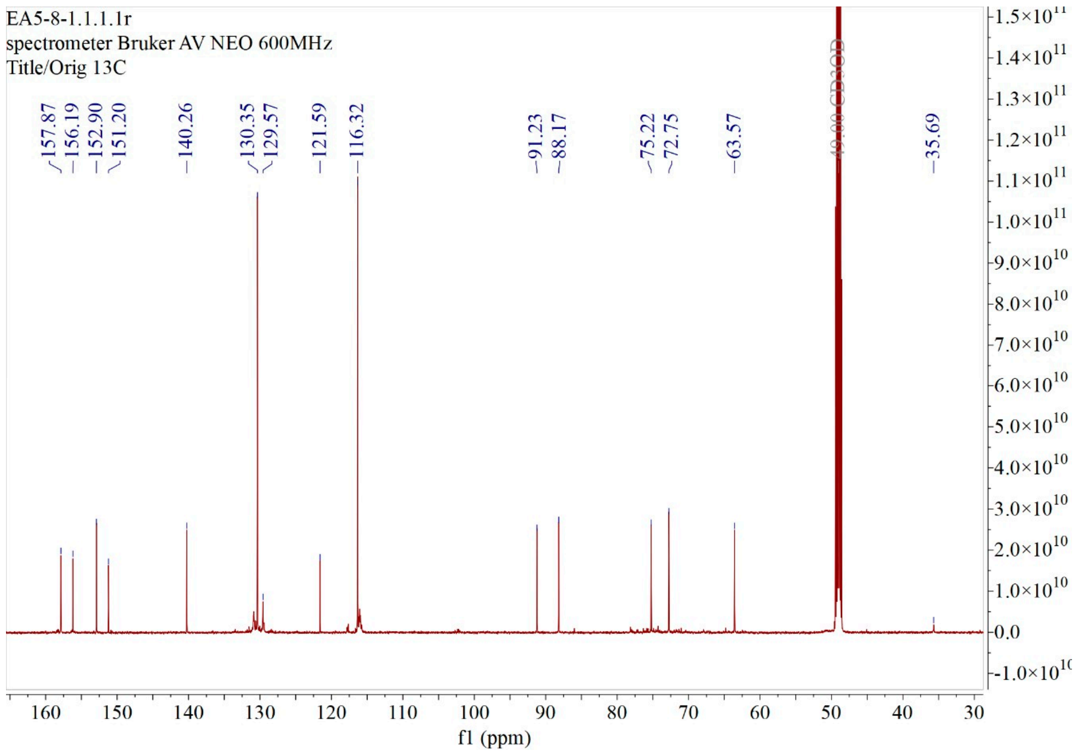

Figure S38. ^{13}C-NMR spectrum of compound **5**, gastronucleoside B (CD_3OD, 150 MHz)

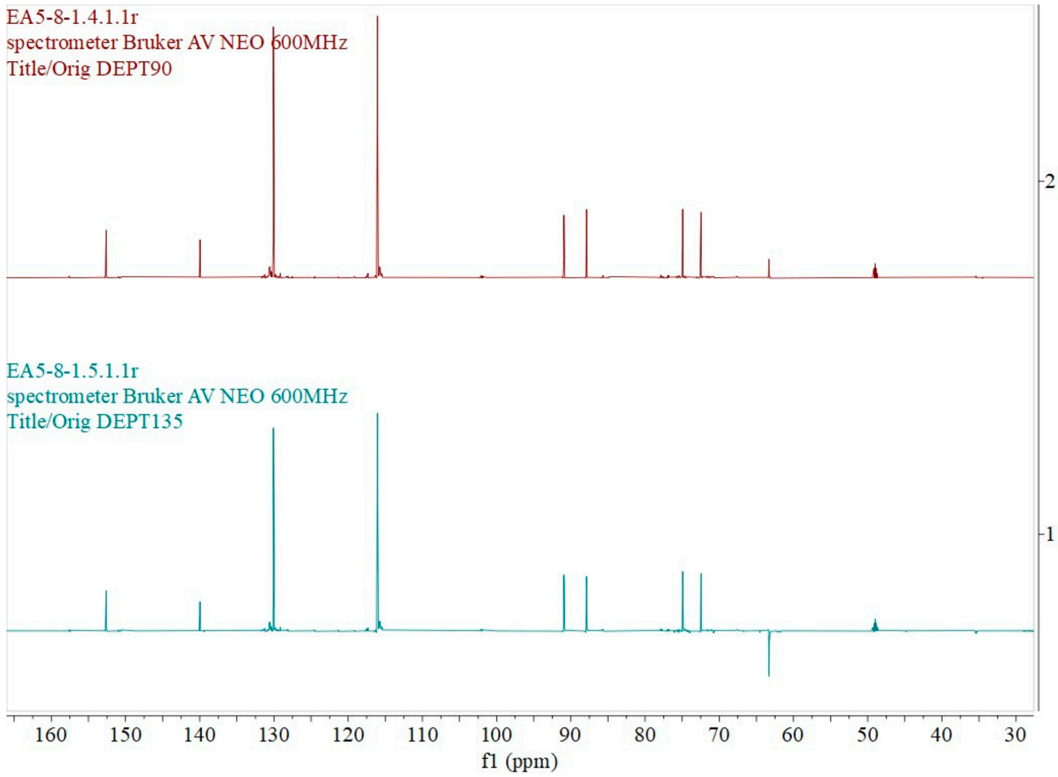

Figure S39. DEPT 90 and 135 spectra of compound **5**, gastronucleoside B (CD_3OD, 150 MHz)

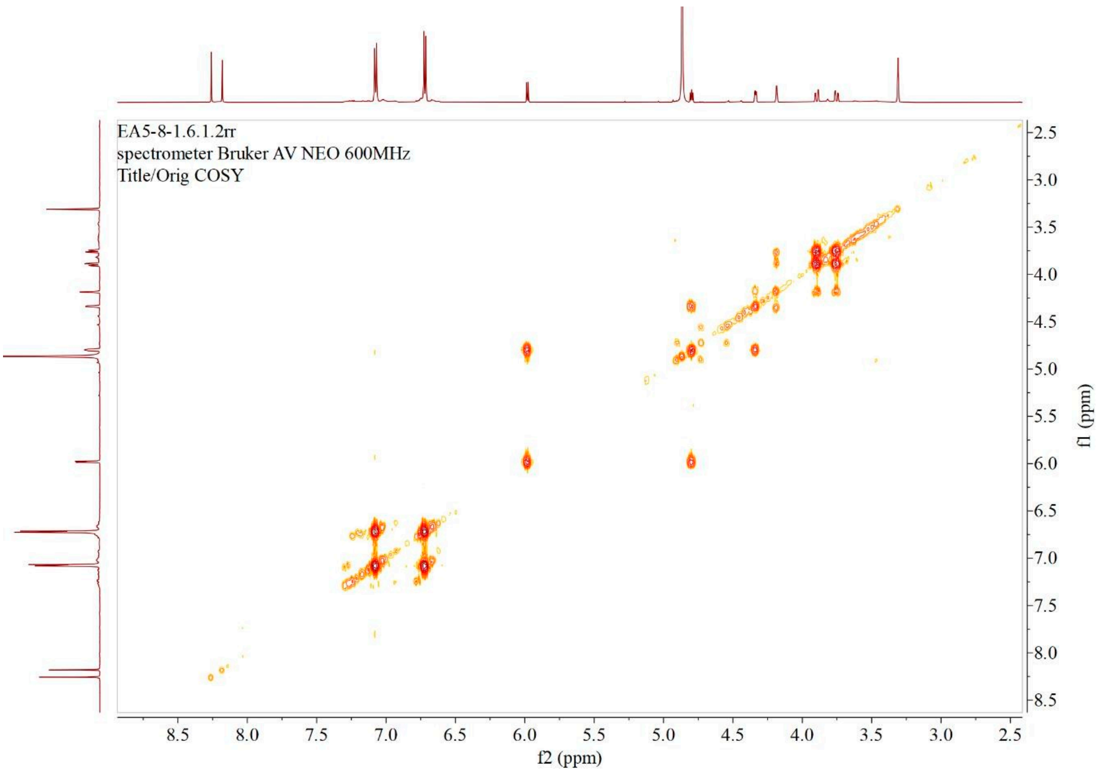

Figure S40. ^{1}H-^{1}H COSY spectrum of compound **5**, gastronucleoside B (CD_3OD)

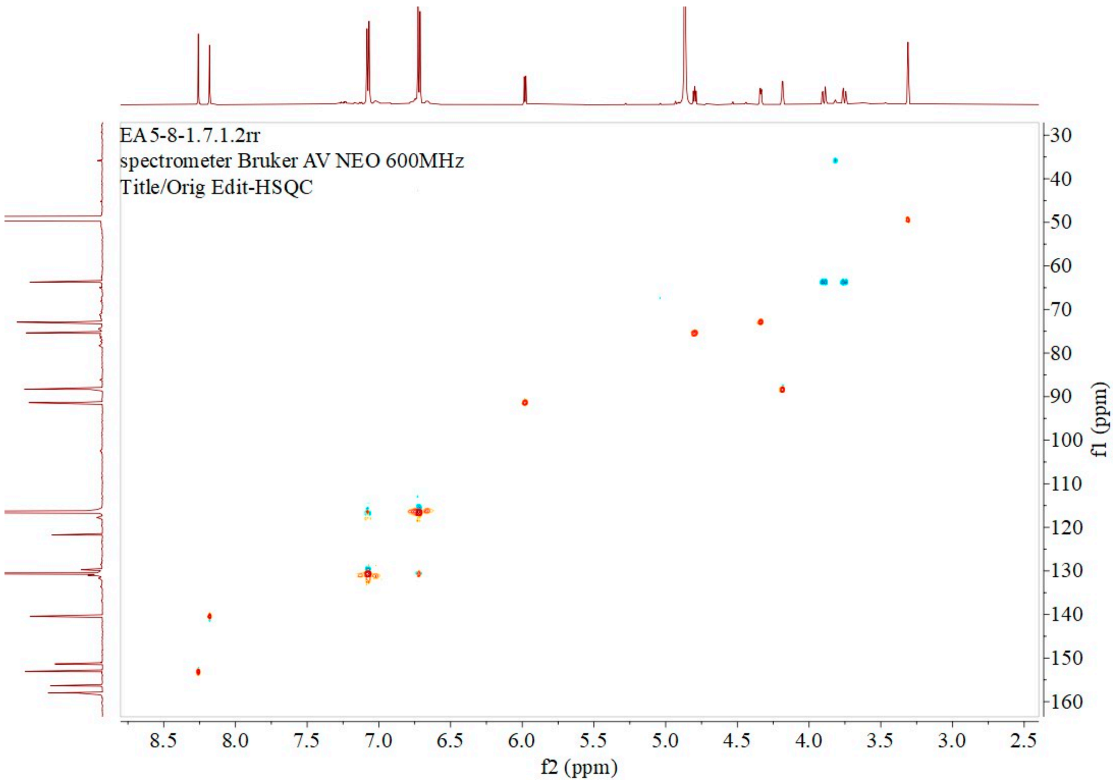

Figure S41. HSQC spectrum of compound **5**, gastronucleoside B (CD_3OD)

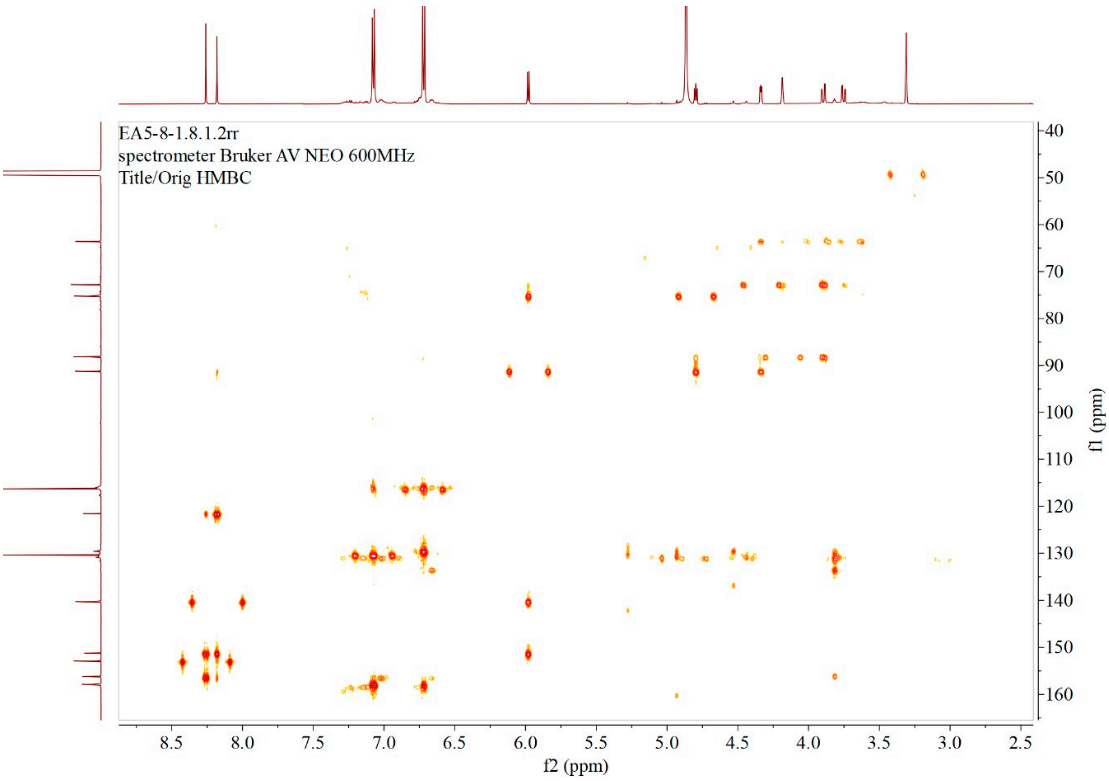

Figure S42. HMBC spectrum of compound **5**, gastronucleoside B (CD_3OD)

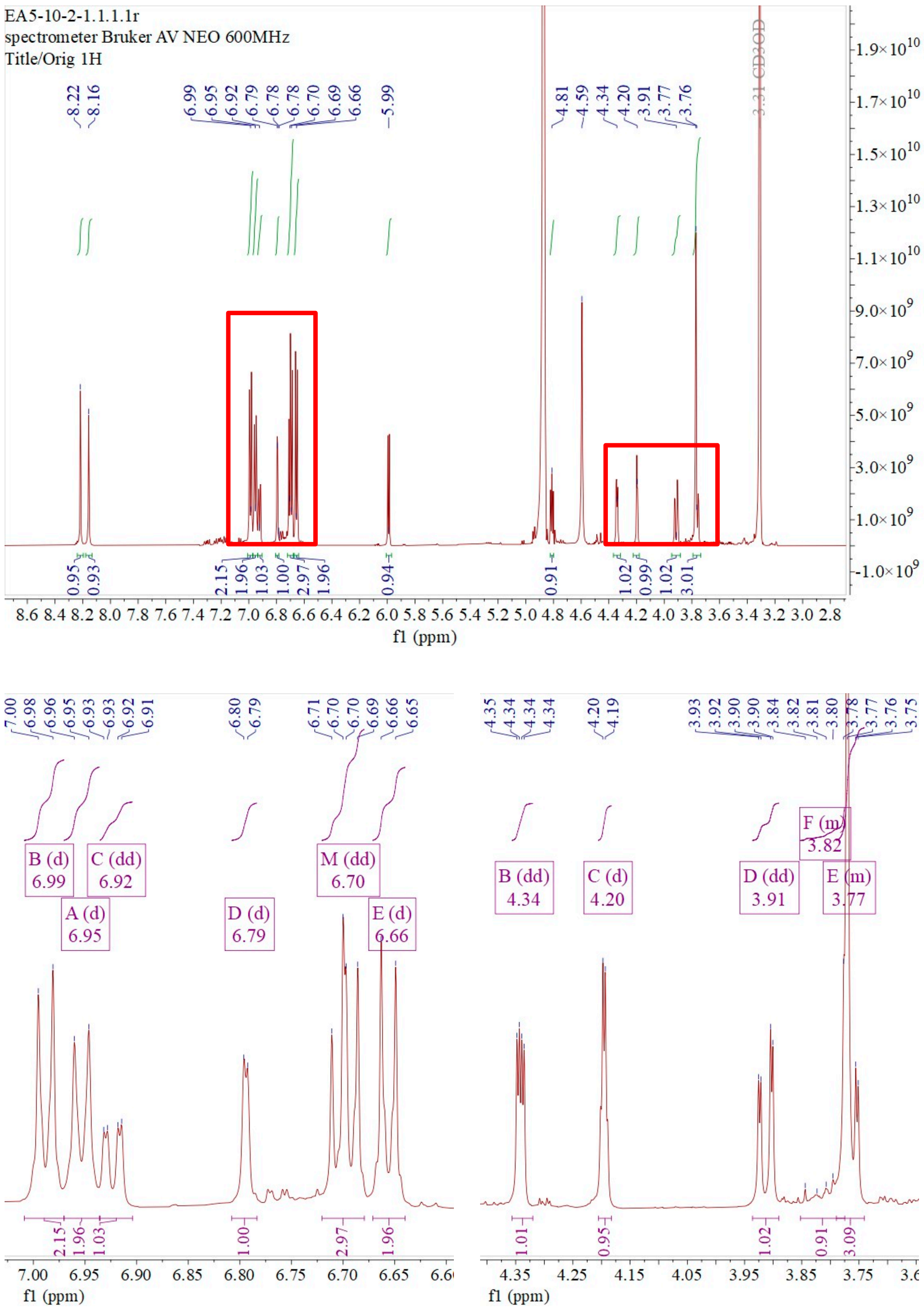

Figure S43. ^{1}H-NMR spectrum of compound **6**, gastronucleoside C (CD_3OD, 600 MHz).

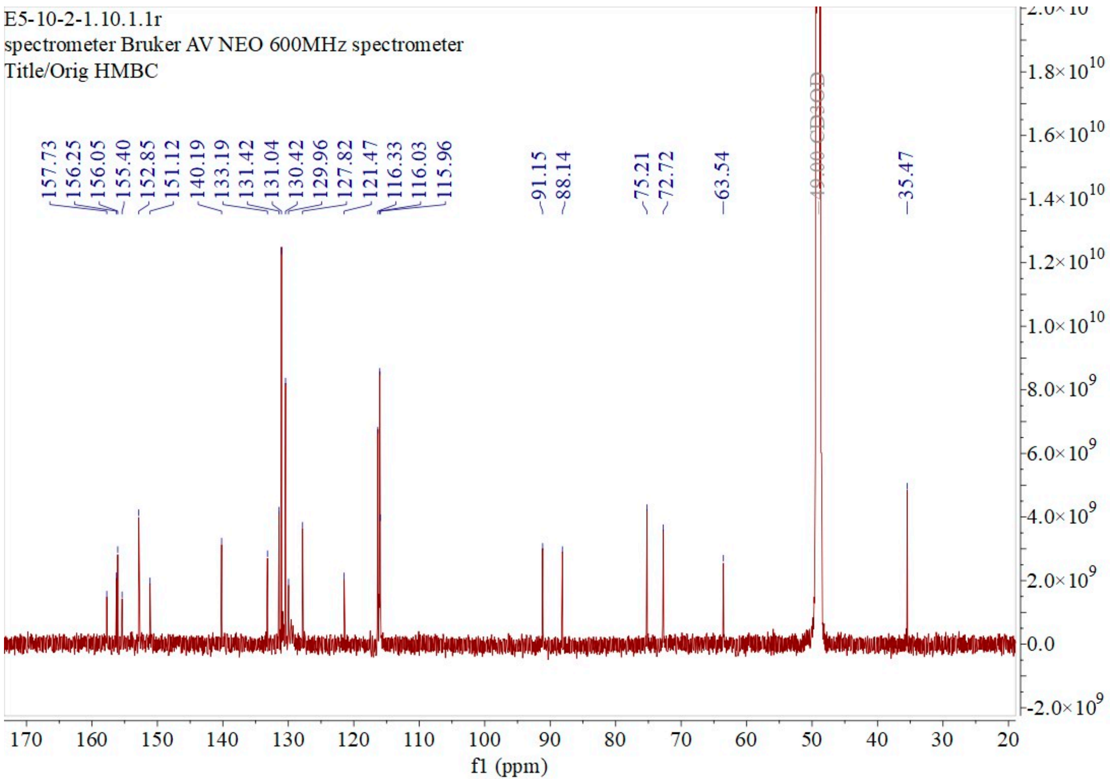

Figure S44. ^{13}C-NMR spectrum of compound **6**, gastronucleoside C (CD_3OD, 150 MHz).

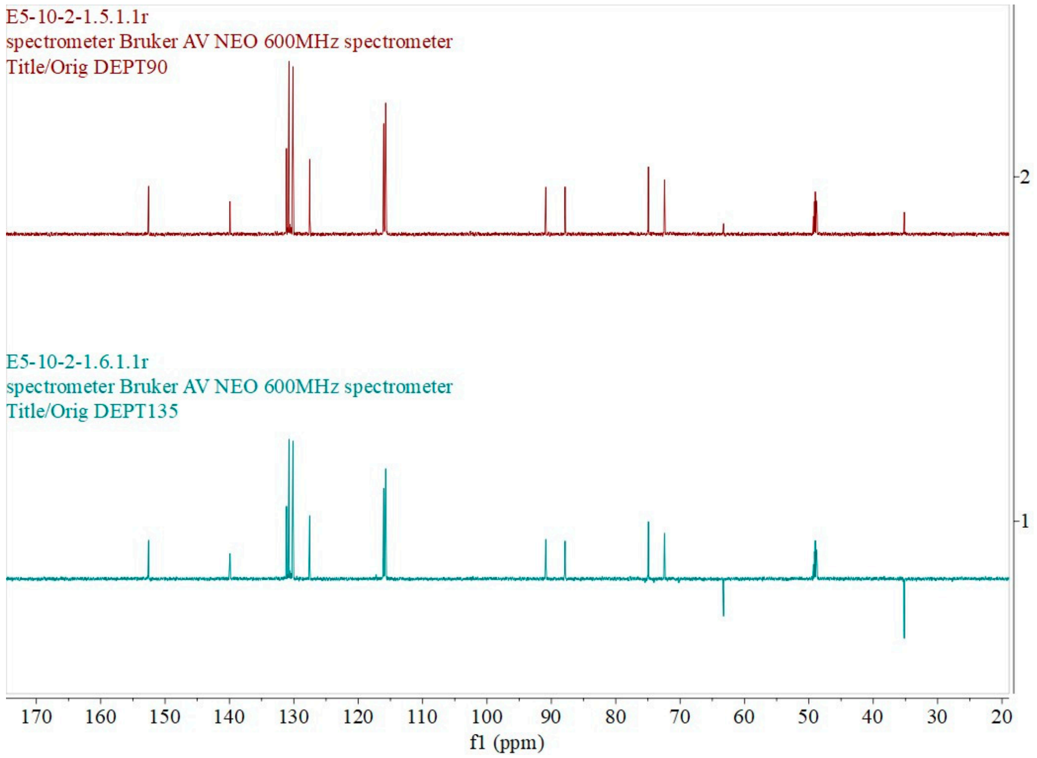

Figure S45. DEPT 90 and 135 spectra of compound **6**, gastronucleoside C (CD_3OD).

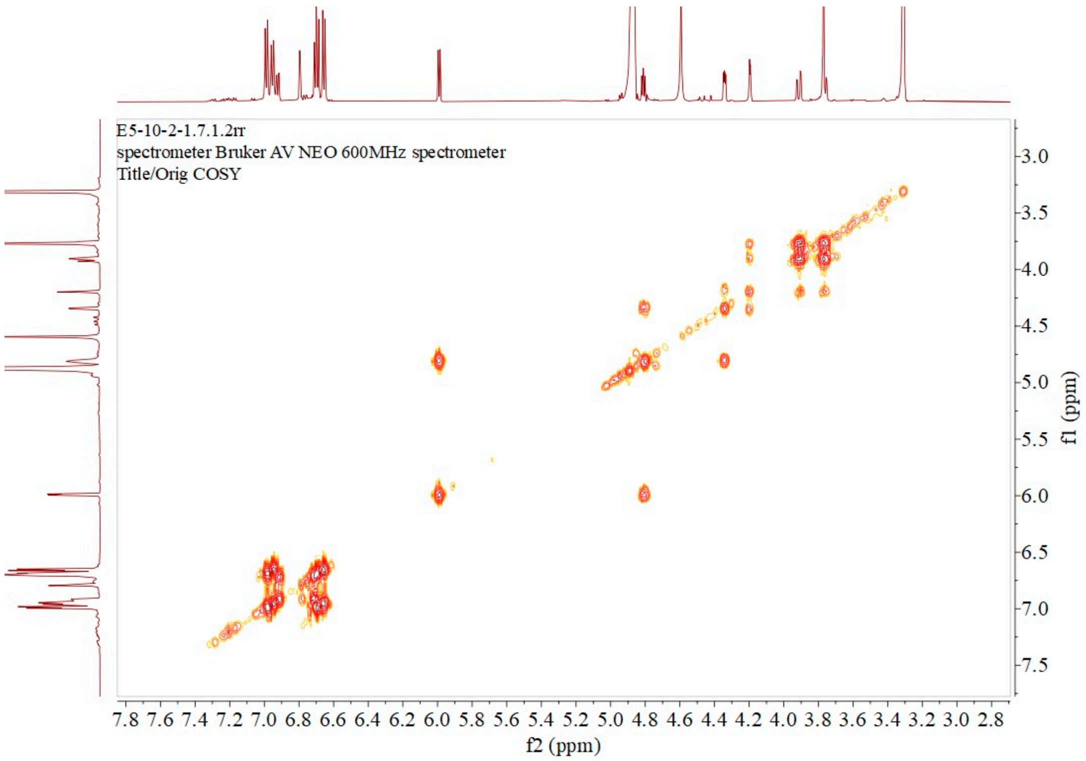

Figure S46. ^{1}H-^{1}H COSY spectrum of compound **6**, gastronucleoside C (CD_3OD).

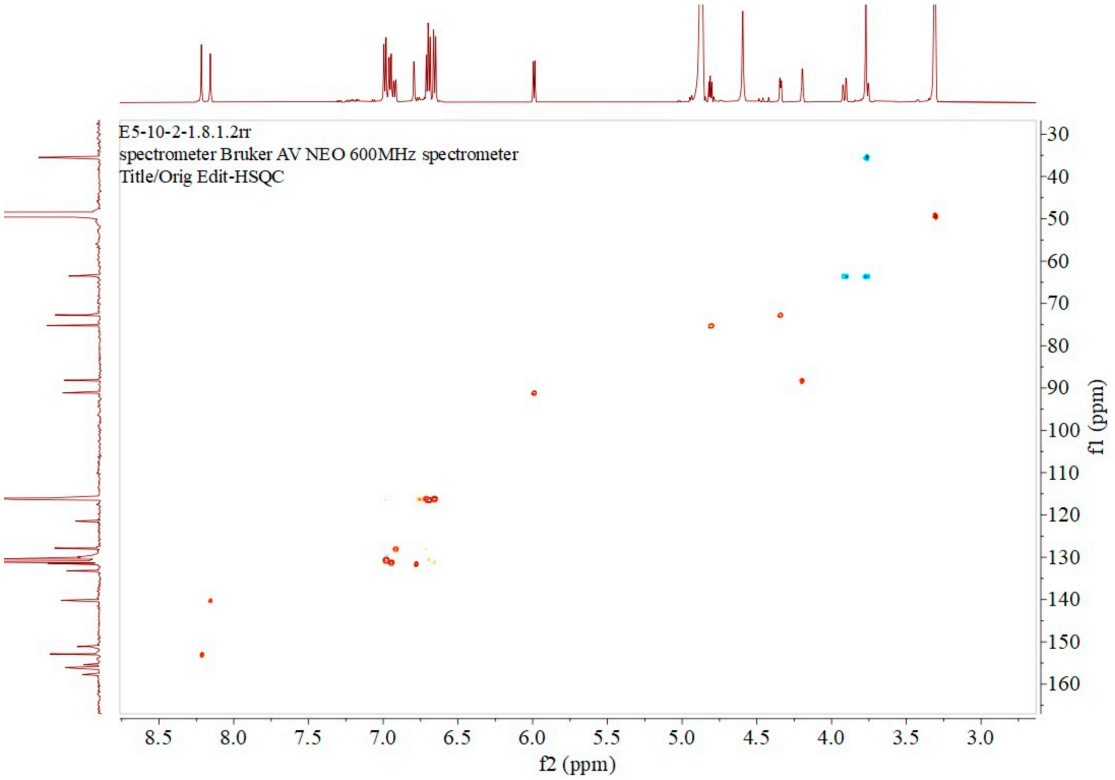

Figure S47. HSQC spectrum of compound **6**, gastronucleoside C (CD_3OD).

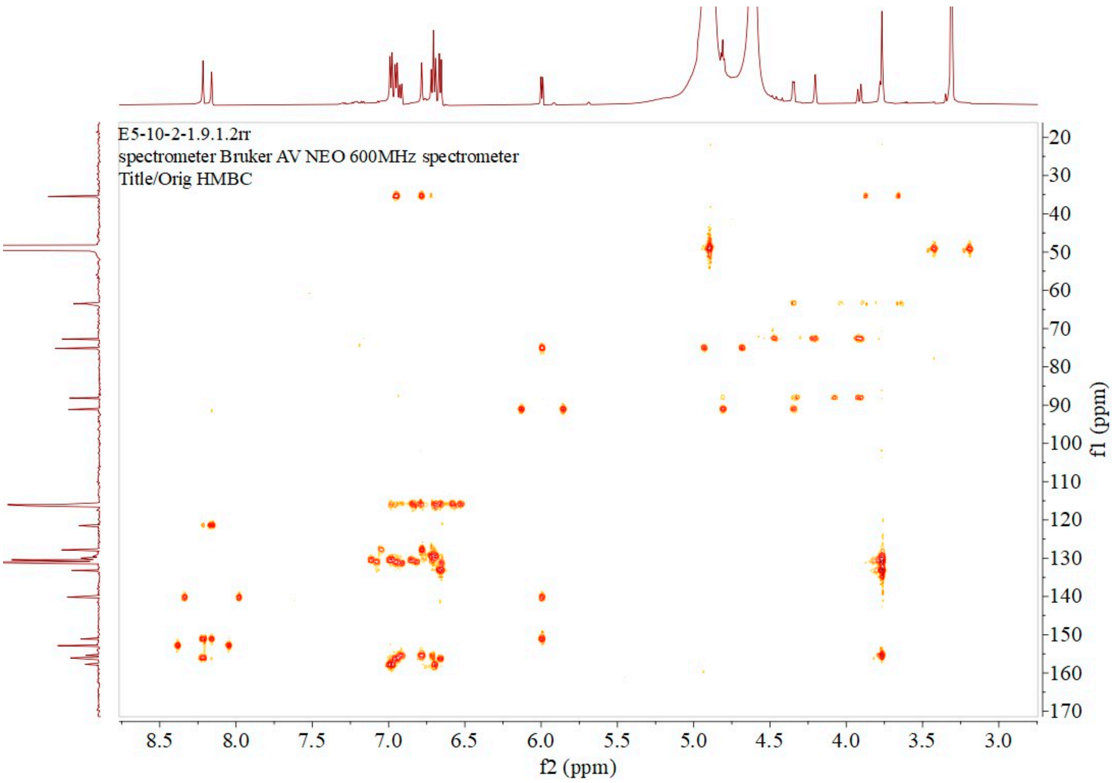

Figure S48. HMBC spectrum of compound **6**, gastronucleoside C (CD_3OD).

Table S1. Cell viability of glutamate-induced HT-22 cells treated by compounds **5** and **11**.

	Blank Control	Model	10 μM Memantine	10 μM **11**	100 μM **11**	250 μM **11**	10 μM **5**	100 μM **5**	250 μM **5**
1	0.955882	50.22%	62.79%	58.38%	61.47%	43.01%	71.10%	65.22%	41.91%
2	0.974265	49.41%	46.62%	59.26%	63.31%	52.50%	65.15%	64.41%	40.51%
3	1.052206	46.84%	56.54%	59.41%	61.99%	49.26%	69.78%	59.41%	44.12%
4	1.018382	52.65%	60.44%	64.12%	62.13%	46.69%	60.59%	59.63%	48.16%
5	0.999265	43.16%	61.91%	55.07%	61.62%	50.07%	67.28%	60.88%	46.91%
Mean	100.00%	48.46%	57.66%	59.25%	62.10%	48.31%	66.78%	61.91%	44.32%
SD	3.77%	3.61%	6.62%	3.24%	0.73%	3.61%	4.15%	2.73%	3.23%

Table S2. Cell viability of glutamate-induced HT-22 cells treated by compounds **1** and **2**.

	Blank Control	Model	10 μM Memantine	10 μM **1**	100 μM **1**	250 μM **1**	10 μM **2**	100 μM **2**	250 μM **2**
1	106.34%	54.96%	56.46%	55.04%	51.15%	49.88%	52.58%	54.48%	54.80%
2	105.63%	54.32%	60.75%	54.48%	49.17%	53.85%	51.63%	55.99%	50.12%
3	94.13%	50.44%	59.64%	55.59%	53.61%	49.01%	51.15%	54.48%	53.53%
4	97.07%	52.82%	60.43%	54.56%	56.94%	48.85%	50.83%	56.30%	50.04%
5	96.83%	53.85%	56.46%	54.88%	52.18%	46.63%	52.82%	54.56%	52.50%
Mean	100.00%	53.28%	58.75%	54.91%	52.61%	49.64%	51.80%	55.16%	52.20%
SD	5.59%	1.77%	2.12%	0.44%	2.91%	2.64%	0.87%	0.91%	2.10%
